# Noise-tolerant LiDAR approaching the standard quantum-limited precision

**DOI:** 10.1038/s41377-025-01790-5

**Published:** 2025-03-26

**Authors:** Haochen Li, Kaimin Zheng, Rui Ge, Labao Zhang, Lijian Zhang, Weiji He, Biao Zhang, Miao Wu, Ben Wang, Minghao Mi, Yanqiu Guan, Jingrou Tan, Hao Wang, Qi Chen, Xuecou Tu, Qingyuan Zhao, Xiaoqing Jia, Jian Chen, Lin Kang, Qian Chen, Peiheng Wu

**Affiliations:** 1 Research Institute of Superconductor Electronics & Key Laboratory of Optoelectronic Devices and Systems with Extreme Performances of MOE, Nanjing University, Nanjing, 210023 China; 2 College of Engineering and Applied Sciences, Nanjing University, Nanjing, 210023 China; 3 Hefei National Laboratory, Hefei, 230088 China; 4 Jiangsu Key Laboratory of Spectral Imaging and Intelligence Sense, Nanjing University of Science and Technology, Nanjing, 210094 China

**Keywords:** Imaging and sensing, Single photons and quantum effects

## Abstract

Quantum-inspired imaging techniques have been proven to be effective for LiDAR with the advances of single photon detectors and computational algorithms. However, due to the disturbance of background noise and the varies of signal in outdoor environment, the performance of LiDAR is still far from its ultimate limit set by the quantum fluctuations of coherent probe light. In this work, we propose and demonstrate a LiDAR from the detection perspective for approaching the standard quantum-limited performance. The photon numbers of echo signals are recorded by a photon-number-resolving detector and applied to overcome heavy background noise through an active photon number filter in the LiDAR. It can approach the standard quantum limit in intensity estimation in a wide photon-flux range, and achieve a Fisher information of only 0.04 dB less than the quantum Fisher information when the mean signal photon number is 10. Experimentally, a noise-free target reconstruction and imaging is demonstrated in the daytime by the proposed LiDAR. It also performs better in reflectivity resolution when taking only 1/1000 of the measurements based on on/off detection. This work provides a fundamental strategy for constructing a LiDAR to quickly extract targets and identify materials in complex environments, which is important for intelligent agents such as autonomous vehicles.

## Abstract (web summary)

A noise-tolerant LiDAR approaching the standard quantum-limited precision through photon-number resolving detection and active photon-number filter.

## Graphical abstract

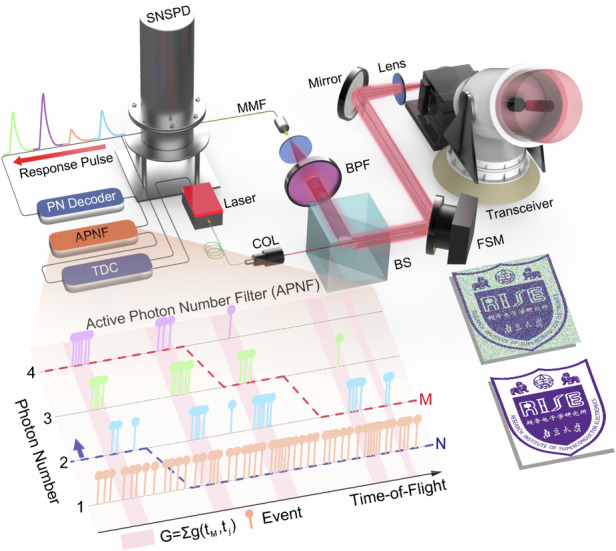

## Introduction

Quantum-inspired technology improves remote sensing and imaging under photon-starved conditions using single-photon LiDAR^1^. It is proven to be an effective method to extract information through computational algorithms when taking the mechanism of single photon detection into account. For example, first-photon imaging can obtain both the depth and reflectivity of the target using only the first detected photon based on the characteristics of low-flux measurement^2^. Computational ghost imaging uses spatial correlations and a single-pixel detector to reconstruct images, which reduces the demand for point-by-point scanning sampling and array detectors^3–6^. Recently, computational imaging has obtained depth images of targets tens or hundreds of kilometers away^7,8^. In addition, non-line-of-sight imaging can also be achieved by reconstructing the transmission path of multiple diffused light^9,10^

An important task of these methods is to extract signal photons and reconstruct targets based on the information encoded in some degrees of freedom of the light field, such as amplitude, frequency^11,12^, phase^13,14^, and polarization^15,16^. In typical single-photon LiDAR, the amplitude of the light field is usually displayed in the form of peaks in temporal statistical histograms instead of the photon number^17^. However, detecting only whether there are photons will lose part of the information under the condition of no scarcity of photons. It prevents measurements from reaching the standard quantum limit (SQL) set by the inherent statistical fluctuation of signal photons and noise photons, which encourages us to find a more efficient detection method^18^. Although coherent detection, such as homodyne detection, has been proven to be noise tolerant^19^ and efficient in reconstructing multiple parameters of light fields^20,21^, it is not suitable for applications when there are only a few signal photons, such as long-range LiDAR.

Photon-number-resolving (PNR) detection can measure the statistical distribution of the light field in a numerical representation, and it is more promising to achieve measurements with robustness to noise and signal strength. For example, in a quantum receiver, the use of PNR can significantly reduce the probability of error in the case of a high mean photon number^22^. At the same time, some quantum metrology applications under a large photon number can also be achieved through PNR detection^20,23,24^. Recently, photon number threshold detection^25^ has been proven to improve the signal-to-noise ratio (SNR) and extract the information of objects of interest^26^ in LiDAR. However, threshold detection may lose the signal events with photons less than the threshold. And a fixed threshold may reduce the detection probability in complex environments where the echo intensity of the target is unknown and varying. As a result, this post-selection process through thresholding may not bring the gain in information due to the reduced probability of successful measurement. Therefore, finding a practical method suitable for arbitrary echo intensity is an urgent task.

In this work, we demonstrate LiDAR detection of outdoor targets through PNR detection with an array superconducting nanowire single photon detector (SNSPD). It approaches the SQL set by the Quantum Fisher information (QFI) in intensity detection. Improvements of 2.35 dB and 33.42 dB in Fisher information (FI) are achieved compared with on/off detection (conventional single photon detection) at mean signal photon numbers of 1 and 10, respectively. Moreover, we propose a practical algorithm called the active photon number filter (APNF) to process the data obtained by PNR detection and experimentally demonstrate a noise-free target reconstruction and imaging in the observation of a steel pylon 900 m away. Combined with Bayesian estimation, the proposed LiDAR can deal with higher reflectivity resolution when taking fewer measurements than on/off detection, and the accuracy of estimation can approach the SQL. We highlight that PNR detection provides more information to overcome noise and accurately reconstruct images. It can increase the interest of intelligent agents, which need quick and accurate detection.

## Results

### Experimental setup

**Fig. 1:**
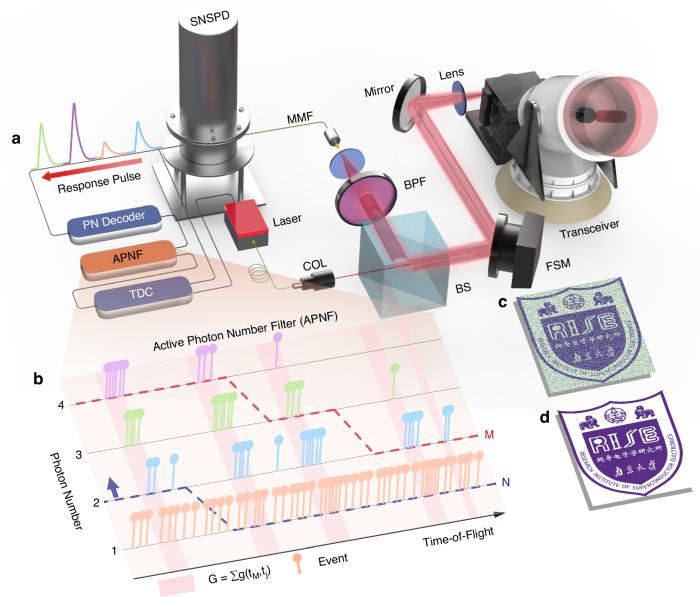
Schematic diagram of the proposed method and simulation of detection results. **a** Schematic diagram of the LiDAR system with PNR. PN Decoder, photon number decoder; APNF, active photon number filter; TDC, time-to-digital converter; COL, collimator; MMF, multimode fiber; BPF, bandpass filter; BS, beam splitter; FSM, fast steering mirror. **b** Schematic diagram of APNF. *M* and *N* represent the chosen maximum and minimum photon numbers, respectively. The deep blue arrow marks the range of photon number determined by *N*. **c** Simulated detection results with on/off detection processed by a log-matched filter. **d** Simulated detection results with PNR detection processed by APNF

A schematic diagram of our method is shown in Fig. 1, and Fig. 1a shows the setup of the system. A collimated pulse laser with a spectrum of 1550 nm and a pulse width of 1 ns is used as the probe light. After passing through a beam splitter (BS), the laser is reflected by a fast-steering mirror (FSM), which is used to scan the target, and then emitted to the target through a transceiver. The transceiver is also used to collect the echo photons. Before being coupled into a multimode fiber (MMF) through a lens, the echo photons are filtered by a bandpass filter (BPF) with a central wavelength of 1550 nm and a bandwidth of 10 nm to reduce background noise. The remaining photons are then detected by an SNSPD array, which can realize quasilinear PNR for up to 16 photons through spatial multiplexing^27^. The SNSPD outputs response pulses with different amplitudes according to photon numbers. The energy of the response pulse is divided into two, with one of them transmitted to the time-to-digital converter (TDC) together with the sync signal from the pulse laser to obtain the time of flight (TOF) and the other transmitted to the photon-number decoder (PN Decoder) to obtain the photon number. Since the power of the noise is uniformly distributed in time while that of the signal is concentrated within the duration of the laser pulse and the events with more photons are more likely to be signal events, we use APNF to extract signals and then reconstruct the target according to the information of flight time and photon number statistics.

A schematic diagram of APNF is shown in Fig. 1b. Different from traditional single-photon LiDAR, we not only record the spatial and temporal information of detection events but also provide a classification according to photon numbers. Two discrimination thresholds, *M* and *N*, are used in APNF. First, the events with at least *N* photons are adopted as effective events for further process. Then we use the flight time of events with at least *M* photons to construct a gate to extract signal events from all effective events. The gate is a set of several subgates and can be expressed as *G* = Σg(*t*_*M*_,*t*_*j*_), while g(*t*_*M*_,*t*_*j*_) represents a subgate whose center is *t*_*M*_ and width is *t*_*j*_. Here, *t*_*j*_ is the time jitter of the whole system, which is mainly determined by the duration time of the pulse laser, and *t*_*M*_ is the flight time of an event with at least *M* photons. The values of *M* and *N* are directly related to the mean signal photon number *μ*_p_. With increasing flight time, which means that the target is farther away from LiDAR, *μ*_p_ as well as the values of *M* and *N* may decrease. To demonstrate the effectiveness of APNF, the Monte Carlo method is used here to generate the arrival of photons and detected events based on on/off detection and PNR detection. Figure 1c, d shows the result with on/off detection processed by the log-matched filter and that with PNR detection processed by APNF, respectively. The result of on/off detection is obviously disturbed by Gaussian noise, while that of PNR detection is not.

### Advantages of APNF

More specifically, the advantages of APNF can be seen from the enhancement of SBR, which is defined by the ratio between signal counts and noise counts^28,29^. The number of detected events can be expressed as the product of the detection probability and the number of measurements. In this work, as long as the detected event contains at least one signal photon, we consider it a signal event. When assuming that the detection efficiency is unity, the SBR of the original detected events for on/off detection can be defined as:

1 $${{\rm{SBR}}}_{{\rm{on}}/{\rm{off}}}=\frac{{\sum }_{i=1}^{f}{\left[1-{p}_{p}\left(0|{\rm{t}}\right)\right]}_{t=i* \triangle t}}{{\sum }_{i=1}^{f}{{p}_{p}(0{|t})[1-{p}_{n}(0)]}_{t=i* \triangle t}}$$

where *f* = *T*_r_*/*Δ*t*, *T*_r_ is the duration time of a measurement which is usually the time interval between laser pulses or the width of temporal gate, and Δ*t* is the duration time of a time bin, which is the same as the system time jitter (1 ns). Thus, *f* is the total number of time bins in which photon detections can be found. In addition, *p*_p_ is the photon number distribution of received probe photons, which follows the Poisson distribution ${p}_{{\rm{p}}}\left(k\right)={\mu }_{{\rm{p}}}^{k}{e}^{{-\mu }_{{\rm{p}}}}/k!$. Similarly, *p*_n_ is the photon number distribution of noise photons mainly coming from the background radiation per Δ*t*, which is usually multimodal for practical LiDAR and follows a Poisson distribution. Here, we ignore the dark counts of approximately 1000 cps (counts per second) coming from the detector since they are much smaller than the level of background noise in this work.

In practice, noise photons arrive in random time, while the signal photons reflected from the target are clustered in the duration time of the laser pulse. When the repetition frequency of the laser pulse is much lower than the noise count rate, there are more noise events than signal events despite *μ*_p_ being larger than the mean noise photon number per time bin *μ*_n_. In this situation, a few signal events may be mixed with many noise events. Thus, it is necessary to build a gate to filter noise. In APNF, assuming *l* laser pulses are taken, the mean number of signal events with at least *N* photons at time bin *t* can be calculated as:

2 $${\left\langle S\right\rangle }_{t}=l\mathop{\sum }\limits_{k=N}^{+\infty }\left[p\left(k|t\right)-{p}_{{\rm{p}}}\left(0|t\right){p}_{{\rm{n}}}\left(k\right)\right]$$

where $p\left({k|t}\right)={\sum }_{m=0}^{k}{p}_{{\rm{p}}}({m|t}){p}_{{\rm{n}}}(k-m)$. Here, ${\left\langle S\right\rangle }_{t}$ can be regarded as the expected intensity of the signal we can obtain. However, there is a probability that we will not detect any events with *m* (*m* ≥ *M*) photons, which will result in a failure in gate construction. Therefore, the final signal intensity should be expressed as:

3 $${S}_{{\rm{APNF}}}=\mathop{\sum }\limits_{i=1}^{f}{\left\langle S\right\rangle }_{t}* {\left[\frac{1-{(1-{\sum }_{k=M}^{+\infty }\,p({k|t}))}^{l}}{1-{(1-{\sum }_{k=N}^{+\infty }\,p({k|t}))}^{l}}\right]}_{t=i* \varDelta t}$$

**Fig. 2:**
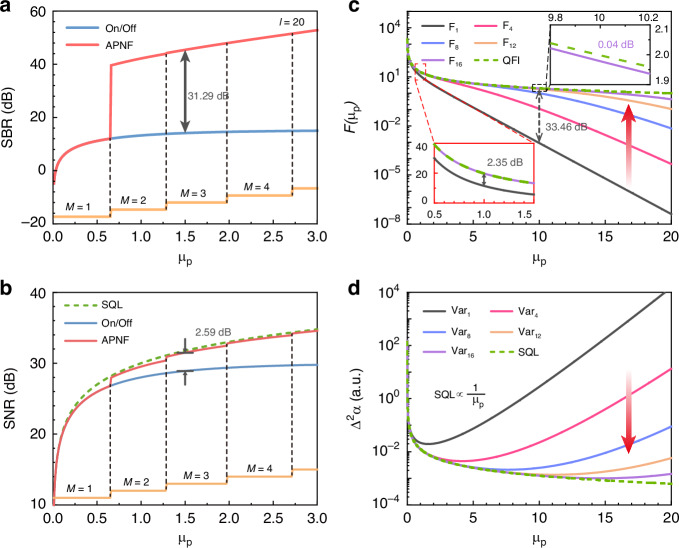
Advantages of PNR detection and APNF when assuming the detector is perfect, *μ*_n_ = 10^–4^ and taking 20 laser pulses. **a** SBR of APNF and on/off detection, where the red line represents SBR_APNF_ and the blue line is SBR_on/off_. The orange line represents the value of *M*. *N* is always 1. **b** SNRs of different methods. **c** FI of *μ*_p_ with different detection methods. *F*_1_, *F*_4_, *F*_8_, *F*_12_ and *F*_16_ represent FIs with detections that can resolve up to 1, 4, 8, 12, and 16 photons. The vertical gray dashed arrow indicates *μ*_p_ = 10, where *F*_1_ is 33.46 dB below the QFI (green dashed line) and that of *F*_16_ is 0.04 dB, as shown in the illustration. When *μ*_p_ = 1, *F*_16_ almost coincides with QFI and is 2.35 dB higher than *F*_1_. **d** ${\Delta}^{2}\alpha$ versus *μ*_p_ when the actual reflectivity $\alpha$ is 0.5. It is calculated from the FI shown in (**c**). The red arrows in (**c**) and (**d**) indicate the variation of FI and ${\Delta}^{2}\alpha$ versus the max resolvable photon number, respectively

The second part on the right is defined as the activation probability, which means the probability of detecting at least one event with more than *M* photons in *l* cycles. In this theoretical calculation, we preset the location of target and set the threshold of the activation probability as 0.95 at the position to avoid failure. Besides, the *S*_APNF_ is expected to be as large as possible (not less than 90% of on/off detection). When meeting these two constraints, we choose the maximum *M* and *N* to ensure that noise can be filtered out (detailed process can be found in Supplementary Information). Then, the intensity of the noise can be calculated as:

4 $${B}_{{\rm{APNF}}}=\mathop{\sum }\limits_{i=1}^{f}{\left\langle B\right\rangle }_{\Delta t}* {\left[\frac{1-{(1-{\sum }_{k=M}^{+\infty }\,p({k|t}))}^{l}}{1-{(1-{\sum }_{k=N}^{+\infty }\,p({k|t}))}^{l}}\right]}_{t=i* \varDelta t}$$

while ${\left\langle B\right\rangle }_{\Delta t}=l* {p}_{p}(0{|t})\mathop{\sum }\nolimits_{k=N}^{+\infty }{p}_{{\rm{n}}}(k)$. Finally, we can take ${{\rm{SBR}}}_{{\rm{APNF}}}={S}_{{\rm{APNF}}}/{B}_{{\rm{APNF}}}$. When assuming *μ*_n_ = 10^-4^, *l* = 20 and the *T*_r_ to be 300 ns according to the experiment, we can obtain the theoretical SBR, as shown in Fig. 2a. The red line and the blue line represent SBR_APNF_ and SBR_on/off_, respectively. The orange line is the value of *M*, while *N* is always 1. When *μ*_p_ is 1.5, an increase of 31.29 dB is observed. This indicates that APNF can effectively filter out noise and extract signals. Besides, the application of PNR detection and APNF can also help to improve the SNR (see *Methods*), as shown in Fig. 2b. When *μ*_p_ is 1.5, the APNF can bring a 2.59 dB advantage compared to on/off detection, and can approach the SQL determined by the fluctuation of photons.

### Information bound of reflectivity estimation

As a key parameter to quantify the performance of LiDAR, SBR is determined by the average level of signal and noise. In practice, the information carried by echo photons is also affected by the statistical fluctuation of the signal and noise, which is what the accuracy of parameter estimation depends on. For example, LiDAR usually reconstructs the reflectivity of a target by normalizing the intensity of detected signals, and the accuracy of intensity estimation can be quantified by FI, which depends on the probe light and detection method. Specifically, the accuracy of intensity estimation can be described by the Cramer-Rao inequality ${\delta }^{2}\left({\mu }_{{\rm{p}}}\right)\ge 1/{lF}\ge 1/l{F}_{Q}$, where *l* is the number of repeated measurements, $F$ is the classical FI of measurement and ${F}_{Q}$ is the QFI determined by the quantum fluctuation of probe light. Therefore, to approach the SQL of reflectivity estimation given by $1/l{F}_{Q}$ with coherent light, the $F$ of measurement needs to be maximized to ${F}_{Q}$ by adopting an optimal detection method^30^, while achieving the Cramer-Rao bound ${\delta }^{2}\left({\mu }_{{\rm{p}}}\right)=1/{lF}$ ^17,31^.

When probe light is emitted by a laser, the reflected field without the presence of background noise can be described by the coherent state $|\Psi \rangle =\hat{D}(\sqrt{{\mu }_{{\rm{p}}}})|0\rangle =\exp \left[\sqrt{{\mu }_{{\rm{p}}}}\left({\hat{a}}^{\dagger }-\hat{a}\right)\right]|0\rangle$. Then the QFI of estimating *μ*_p_ from $|\Psi \rangle$ can be calculated as:

5 $${F}_{{\rm{Q}}}=4\left(\left\langle {\partial }_{{\mu }_{{\rm{p}}}}\varPsi |{\partial }_{{\mu }_{{\rm{p}}}}\varPsi \right\rangle -{\left|\left\langle {\partial }_{{\mu }_{{\rm{p}}}}\varPsi |\varPsi \right\rangle \right|}^{2}\right)=\frac{1}{{\mu }_{{\rm{p}}}}$$

For the case in which the echo light is detected with a PNR detector in the presence of noise, the probability distribution of detection can be expressed as:

6 $${p}_{{\rm{PNR}}}\left(k|{\mu }_{{\rm{p}}}\right)=\left\{\begin{array}{l}p\left(k\right)\qquad\qquad\quad\,{k} < {N}_{0}\\ 1-{\sum }_{m=0}^{{N}_{0}-1}p\left(m\right)\,k={N}_{0}\end{array}\right.$$

where *N*_0_ is the maximum resolvable number of photons, $p\left(k\right)=p\left({k|t}\right)$ mentioned above. When *N*_0_ is 1, it is equal to on/off detection. The corresponding classical FI obtained by PNR detection can be calculated as^23,30^:

7 $${F}_{{N}_{0}}=\sum {\left(\frac{\partial {lnp}\left(k|{\mu }_{p}\right)}{\partial {\mu }_{p}}\right)}^{2}p\left(k|{\mu }_{p}\right)$$

When taking *μ*_n_ = 10^-4^ and assuming unity detection efficiency, ${F}_{{N}_{0}}$ with *N*_0_ = 1, 4, 8, 12, 16 versus *μ*_p_ is shown in Fig. 2c. The green dashes represent the QFI, while other ${F}_{{N}_{0}}$ are marked with different colors. To be consistent with Fig. 2a, we multiply all the results by 20, which is the number of repeated measurements. It shows that ${F}_{{N}_{0}}$ increases with increasing *N*_0,_ and when *μ*_p_ = 10, *F*_16_ is only 0.04 dB lower than QFI, while that of *F*_1_ is 33.46 dB. When *μ*_p_ = 1, *F*_16_ almost coincides with QFI, and *F*_1_ is 2.35 dB below them. When taking 3 dB lower than QFI as a reference, the dynamic range of *F*_16_ is 11.84 dB greater than that of *F*_1_ (Supplementary Information). The FI of *μ*_p_ can indirectly give the precision bound of reflectivity estimation. Compared with on/off detection, the use of PNR is helpful for improving the FI, and the reconstruction accuracy of the target’s reflectivity can also be improved. Figure 2d gives the variance of reflectivity measurement (see *Methods*) when assuming the actual reflectivity $\alpha$ is 0.5. The greed dashed line is calculated from the QFI shown in Fig. 2c and scales with $1/{\mu }_{{\rm{p}}}$, indicating the standard quantum limit (SQL). *Var*_1_ to *Var*_16_ corresponds to the ${\triangle }^{2}\alpha$ with detections which resolve up to 1 to 16 photons. It can be seen that the *Var*_1_ deviates the SQL rapidly with the increase of ${\mu }_{p}$, while the results of PNR detection are in line with the SQL in larger dynamic ranges. The red arrows in Fig. 2c, d indicate that FI increases with increasing max resolvable photon number, whereas ${\triangle }^{2}\alpha$ decreases with it, respectively. These results prove the enhancement of PNR detection on LiDAR, especially approaching the standard quantum-limited precision in reflectivity measurement. We also analyze the information bound of depth estimation in Supplementary Note 22.

### Extracting the signal with APNF

The detection target is a distributed steel pylon^32^ with a material size of ~10 cm located approximately $900\,{\rm{m}}$ away. In this experiment, the laser repetition frequency is 20 kHz with a pulse width of 1 ns, and the single pulse energy is 14 μJ. The step value of the FSM is set to 0.003° with a scanning range of $\pm$0.3°. In the case of high-speed detection, it is an extremely heavy task for the signal acquisition system to record the amplitude and time simultaneously. Therefore, we use different photon number thresholds to record events. We find that the events mainly contain one to four photons, and those with more photons can be neglected. The count rate coming from background noise is ~10^5 ^cps even when filtered by the BPF.

**Fig. 3:**
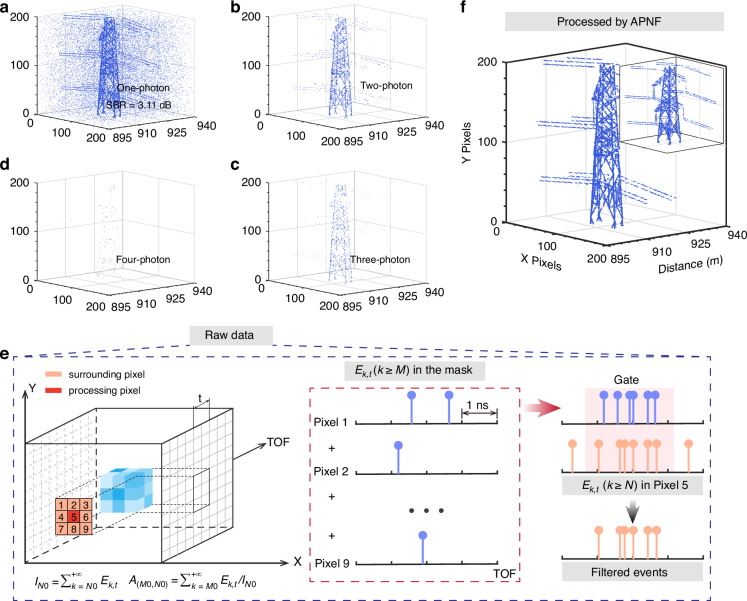
Experimental results and schematic diagram of data processing with APNF. **a**–**d** Raw data obtained with a one-photon to four-photon threshold, respectively. **e** Schematic of the practical algorithm. A 3D mask is used to traverse the raw data, and the intensity *I*_*N*0_ of events with at least *N*_0_ photons as well as the ratio *A*_(*M*0, *N*0)_ of at least *M*_0_ photons can be calculated. The selected *M* and *N* are the max values of *M*_0_ and *N*_0_ meeting ${I}_{N0}\ge 0.9\times {\sum }_{k=1}^{+\infty }{E}_{k,t}$ and ${A}_{\left(M0,N0\right)}\ge 1-{0.05}^{1/l}$. Then, the events with at least *M* photons in the mask are used to filter the events with at least *N* photons in the processing pixel. **f** The result is processed by APNF. The inset shows the details of the pylon

The experimental results and schematic diagram of data processing with APNF are shown in Fig. 3. We emit 20 laser pulses in 1 ms and record the detection events with one-photon to four-photon threshold during this integration time, corresponding to 20 repeated measurements per pixel (mpp), as shown in Fig. 3a–d. The total acquisition time to measure the target is 40 s. These results are obtained by applying a 300 ns width global temporal gate to subtract most noise. And the original results are provided in the Supplementary Information. The signal events obtained by on/off detection (one-photon threshold) are flooded by enormous noise in Fig. 3(a). With the threshold increase to two, the outline of the target can be observed. However, the signal has been lost considerably when the threshold is larger than two, although the noise is also reduced. Here, the APNF is benefit for avoiding this problem brought about by threshold detection. A schematic diagram of raw data processing with APNF is shown in Fig. 3e. A 3D mask is used in APNF to select *M* and *N* as well as construct pixelwise gate for filtering. Detailed descriptions can be seen in “*Methods*”. Figure 3f shows the output of APNF in which there are nearly no noise events can be observed and the inset gives the details. This result clearly shows the structure of the pylon, indicating the capability of APNF to extract signals from noise. The results of original data without the global gate are also provided in Supplementary Note 18. We also compare the results processed by APNF and threshold detection to show the advantages of APNF, and details can be found in Supplementary Note 5.

### Reflectivity estimation

According to the LiDAR equation (Supplementary Information), the intensity of the received signal is linearly correlated with the target’s reflectivity. The relative reflectivity of the target can be accessed by normalizing *μ*_p_. Here, we introduce Bayesian estimation to estimate *μ*_p_. The estimator of *μ*_p_ based on *n* repeated measurements can be derived from the probability:

8 $$p\left({\mu }_{{\rm{p}}}|{k}_{1}\ldots {k}_{n}\right)=\frac{p\left({\mu }_{{\rm{p}}}\right)p\left({k}_{1}|{\mu }_{{\rm{p}}}\right)\ldots p\left({k}_{{\rm{n}}}|{\mu }_{{\rm{p}}}\right)}{\int p\left({\mu }_{{\rm{p}}}\right)p\left({k}_{1}|{\mu }_{{\rm{p}}}\right)\ldots p\left({k}_{{\rm{n}}}|{\mu }_{{\rm{p}}}\right)d{\mu }_{{\rm{p}}}}$$

where *k*_n_ represents that the detector has detected *k* photons in the *n*th detection, and $p\left({\mu }_{{\rm{p}}}\right)$ is the prior probability of *μ*_p,_ which is equal for any *μ*_p_ and can be considered a constant. $p\left({k|}{\mu }_{{\rm{p}}}\right)$ is the conditional probability, which can be calculated as eq. S3 in the Supplementary Information. We take the estimated intensity as ${\hat{\mu }}_{{\rm{p}}}=\sum {\mu }_{{\rm{p}}}p({\mu }_{{\rm{p}}}{|k})$ and take its variance as ${\delta }^{2}\left({\hat{\mu }}_{{\rm{p}}}\right)=\sum {\mu }_{{\rm{p}}}^{2}p({\mu }_{{\rm{p}}}{|k})-{\hat{\mu }}_{{\rm{p}}}^{2}$.

**Fig. 4:**
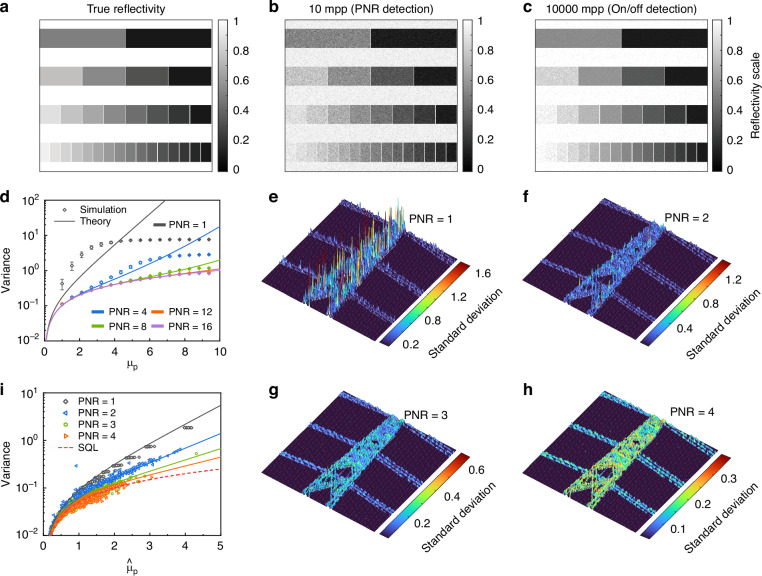
Reflectivity estimation. **a** The grayscale image used in Monte Carlo simulation. The maximum mean signal photon number is set to 10. **b** The estimated results are based on 10 measurements per pixel (mpp) with PNR detection, which can resolve up to 16 photons. **c** The estimated results based on 10000 mpp with on/off detection. **d** The variances of the simulated estimation. **e**-**h** Standard deviation (Std) of experimental estimation of ${\hat{\mu }}_{{\rm{p}}}$ with different PNRs. PNR = 1 to PNR = 4 indicates that the detector can resolve up to 1 to 4 photons. The height and color represent the value of Std. **i** The dots represent variances of experimental estimation, and the lines are theoretical curves

We use a linear grayscale image to test the reflectivity estimation capability of our method, as shown in Fig. 4a. The image has a 1-bit to 4-bit grayscale, corresponding to 2 to 16 reflectivity levels. The Monte Carlo process is used to generate detected events based on PNR detection and on/off detection. We assume that the maximum detected mean photon number is 10 and the detection efficiency is unity. Figure 4b shows the estimated result based on PNR detection, which can resolve up to 16 photons. It can successfully resolve 16 levels based on 10 mpp, while on/off detection needs 10000 mpp to resolve 15 levels and fails to resolve the last one, as shown in Fig. 4c. The variances of estimation based on different detection methods when applying 10 mpp are shown as hollow points in Fig. 4d. The solid lines are the theoretical results calculated from the FI. The variance of on/off detection (PNR = 1) deviates from the theoretical line, indicating a biased estimation. In addition, that of PNR = 16 is consistent with the theoretical prediction, indicating an unbiased estimation.

Experimentally, we extract the events with different photon numbers in each spatial pixel of Fig. 3f. And the number of events is corrected by the positive-operator-valued measure (POVM) elements of the detector. Then, Bayesian estimation is used to obtain ${\hat{\mu }}_{{\rm{p}}}$ and the corresponding variances. Figure 4e–h shows the standard deviation (Std) of the estimation of ${\hat{\mu }}_{{\rm{p}}}$ with different PNRs. The height and color represent the value of Std, and the fluctuation of height reveals the fluctuation of Std. Std and its fluctuation decrease with the increase in PNR capability, which indicates a more accurate and more robust estimation. Figure 4i shows the variances of the experimental results and their theoretical curves. The red dashed line represents the SQL. The trends of the experimental results are close to the theoretical lines, indicating a near unbiased estimation. The results of PNR = 4 approach the SQL, which indicates that the estimation can approach the standard quantum-limited precision. The spatial distribution of estimated ${\hat{\mu }}_{{\rm{p}}}$ is provide in the Supplementary Note 11. The ${\hat{\mu }}_{{\rm{p}}}$ of the echo signal from the main body of the pylon is relatively large, most distributing between 1 and 2. But those of the echo signal from the two side cables is smaller, around 0.5, and even around 0.1 at some regions. It precisely reflects the wide distribution of signal intensity in outdoor environment, which makes traditional single photon LiDAR unable to constantly maintain high measurement accuracy. In both low and high photon flux regime, PNR detection can perform better than on/off detection, increasing the dynamic range of LiDAR to work in complex environments.

### Noise-tolerant reconstruction

**Fig. 5:**
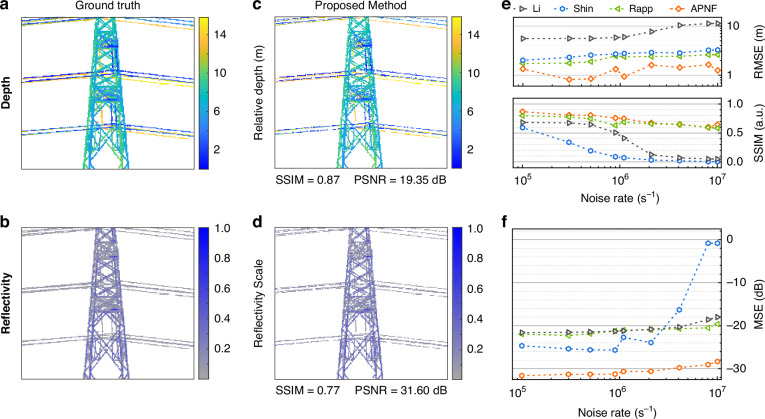
Noise-tolerant reconstruction. **a**, **b** The ground truth obtained by long-time detection. The white regions represent null values. **c**, **d** The reconstructed depth image with maximum-likelihood estimation and the reconstructed reflectivity image with Bayesian estimation based on the data shown in Fig. 3f. **e**, **f** Comparison with other computational algorithms used in single-photon LiDAR when applying different noise rates

Based on the detected events, the reflectivity and depth images of the pylon can be reconstructed. The results obtained by long-time detection are used as references (ground truth), as shown in Fig. 5a, b. Using the data shown in Fig. 3f, we reconstruct the depth and reflectivity images through maximum-likelihood estimation and Bayesian estimation, respectively. The results can be seen in Fig. 5c, d. Structure similarity (SSIM) and peak signal-to-noise ratio (PSNR), which are parameters to weigh the difference from the reference, are used to judge the quality of these images. They all reach relatively high values, which indicates that the images are well reconstructed. To test the noise tolerance of the proposed method, we add noise following the Poisson distribution into the raw data through Monte Carlo. Then, we use APNF to process the noise-enhanced data and reconstruct the images. Here, we also use another computational algorithm used in single-photon LiDAR as a reference. The root mean square error (RMSE) and SSIM compared to the ground truth of the depth image are shown in Fig. 5c. As the noise rate increases from 10^5^ to 10^7 ^s^−1^, the RMSE of APNF does not increase substantially and remains lower than that of the other three methods. For SSIM, the performance of APNF is close to that of Rapp^29^ and is much better than the other two. The mean square error (MSE) of the reflectivity image is shown in Fig. 5f. Although the MSE of APNF increases with the noise rate, it is always ~10 dB lower than Li^7^ and Rapp. The MSE of Shin^31,33^ increases rapidly when the noise rate is larger than 10^6 ^s^−1^. These results prove the noise-tolerant capability of the proposed method. The details of the reconstruction can be found in the Supplementary Information.

## Discussion

In summary, a noise-tolerant LiDAR towards the standard quantum-limited precision in terms of intensity is proposed here. In combination with photon-number-resolving detection and an active photon number filter (APNF), the LiDAR demonstrates an increased dynamic range and an improved capability to detect targets in heavy noise. We experimentally detect a steel pylon 900 m away and accurately observe its 3D structure through the point cloud processed by APNF. A noise-free target reconstruction and imaging in daytime is achieved in practice. In addition, more accurate reflectivity estimation based on fewer measurements compared with traditional on/off detection is demonstrated using Bayesian estimation. These improvements stem from the increase in information obtained by detection strategy. It can realize remote sensing and image reconstruction in complex environments. We believe that this work provides a way to apply photon-sensitive LiDAR to autonomous vehicle detection and target recognition, which requires all-time operation and quick detection.

## Materials and methods

### Readout and acquisition of photon number resolving pulses

The SNSPD array used in this work has 16 pixels and can realize quasilinear PNR for up to 16 photons through spatial multiplexing. In practice, each pixel works independently. Therefore, 16 readout circuits are needed to read the output pulses of different pixels. When multiple photons are incident, each photon may be absorbed by a pixel. The pixels that have absorbed at least one photon have a certain probability of outputting a pulse, while the probability is the quantum efficiency of the pixel. We use low noise amplifiers (mini-circuits LNA 650) to readout the pulses of different pixels and then use a power combiner (mini-circuits ZC16PD-222-S+) to combine the pulses’ energy. This will result in new pulses with different amplitudes according to different numbers of fired pixels. The amplitude of the pulse can be recorded by a high-speed oscilloscope (Keysight MSOV334A), while the time-of-flight is read through a 4-channel time-correlated single photon counter (TCSPC, HydraHarp 400). In the experiment, we can divide the energy of the combined pulse into four, and each of them is transmitted to a channel of the TCSPC. The discrimination thresholds of the TCSPC’s 4 channels are set to be different, corresponding one-photon to four-photon. Thus, the number of events with different photon numbers can be obtained.

### SNR improvement with APNF

Considering that though the improvement of SBR shows the advantage in processing noise-mixed data and acquiring high-quality results, it is not sufficient in expressing the sensitivity of the LiDAR. We further analyze the improvement of SNR brought by APNF, which can represent the sensitivity of LiDAR to reflected signal. First, we consider the perfect intensity detection which can directly show the intensity of signal and noise. When applying *l* times perfect intensity detection, the shot-noise limited SNR determined by the fluctuation of photons can be expressed as^34^:

$${{\rm{SNR}}}_{{\rm{Q}}}=\frac{l{\mu }_{{\rm{p}}}}{\sqrt{l{\mu }_{n}}}$$

where $l{\mu }_{{\rm{p}}}$ is the height of the signal peak and $\sqrt{l{\mu }_{n}}$ is the noise floor. However, due to the quantization of the light, traditional intensity detection is not applicable when there are only several photons. In this case, photon-click detection, such as on/off detection and PNR detection, is usually used. For on/off detection, the SNR can be calculated as:

$${{\rm{SNR}}}_{{\rm{on}}/{\rm{off}}}=\frac{l\left({p}^{{\prime} }-{p}_{n}^{{\prime} }\right)}{\sqrt{l\left[{p}_{n}^{{\prime} }\left(1-{p}_{n}^{{\prime} }\right)\right]}}$$

where ${p}^{{\prime} }=1-p(0)$ is the probability to detect an event, ${p}_{n}^{{\prime} }=1-{p}_{{\rm{n}}}(0)$ is the probability to detect noise photons. And $p\left(k\right)$ is the convolution of signal and noise as defined in the main text. For on/off detection, the output is 0 or 1 for each detection. But PNR detection can output the number of photons. So that, when using PNR detection and adopting the events with *N* to *M* photons, the SNR is:

$${{\rm{SNR}}}_{{\rm{APNF}}}=\frac{l\left[{\sum }_{k=N}^{M-1}\,k\rho (k)+M{\sum }_{k=M}^{+\infty }\,\rho (k)\right]}{\sqrt{l\times \mathrm{var}(N)}}$$

${\mathrm{var}}\left(N\right)={\sum }_{k=N}^{M-1}\,{k}^{2}{p}_{n}\left(k\right)+{M}^{2}{\sum }_{k=M}^{+\infty }\,{p}_{n}\left(k\right)-{\left[{\sum }_{k=N}^{M-1}\,k{p}_{{\rm{n}}}\left(k\right)+M{\sum }_{k=M}^{+\infty }\,{p}_{n}(k)\right]}^{2}$ represents the variance of the noise, $\rho \left(k\right)=p\left(k\right)-{p}_{n}(k)$, *N* and *M* are determined by APNF. Different from the SBR which only consider the number of events, SNR takes each detected photons into account. So that *k* detected photons means *k* signals or noise. And the events with more than *M* photons are regards as the events with *M* photons. The improvement of SNR is beneficial for both extracting the target and increasing the accuracy of ranging.

### Variance of reflectivity measurement

According to the LiDAR equation given in the Supplementary Information eq. S1, the relationship between *μ*_p_ and reflectivity *α* can be written as μ_p_ = *Cα*, where *C* is the detection resource which can be regarded as the average number of photons the LiDAR received when the reflectivity is unity, and it is determined by the distance of the target, parameters of LiDAR system and the transmission of atmosphere. Then the variance of *α* measurement can be calculated as:

$${\triangle }^{2}\alpha =\frac{{\triangle }^{2}{\mu }_{p}}{{C}^{2}}=\frac{{\alpha }^{2}}{{\mu }_{p}^{2}{lF}\left({\mu }_{p}\right)}$$

where *l* is the measurement time (the number of laser pulses we used to measure) and $F\left({\mu }_{p}\right)$ is the FI of ${\mu }_{p}$. And ${\triangle }^{2}{\mu }_{p}=1/{lF}\left({\mu }_{p}\right)$ is determined by the Cramer-Rao law. When considering the QFI $F=1/{\mu }_{p}$, the equation is converted to be ${\triangle }^{2}\alpha ={\alpha }^{2}/l{\mu }_{p}$. It can be seen that the variance for a given $\alpha$ scales with $1/{\mu }_{p}$, indicating the standard quantum limit (SQL).

Besides calculating the variance, the performance of LiDAR in detection and imaging can also be quantified by the Shannon information inferred from the FI. The details of the Shannon information model can be found in the Supplementary Information.

### Details of the practical APNF

The 3D mask in Fig. 3e is composed of 9 pixels and has a duration time of 4 ns. The red pixel (pixel 5) is the processing pixel, and the surrounding pixels (pixels 1-4&6-9) are used as references. Within each 3D mask, we count all events with at least *N*_0_ photons, defined as ${I}_{N0}={\sum }_{k=N0}^{+\infty }{E}_{k,t}$ in Fig. 3e, while ${E}_{k,t}$ represents the events with *k* photons in time *t*. Then, we calculate ${A}_{(M0,N0)}$, which is defined as the ratio between ${I}_{M0}$ and ${I}_{N0}$. The values of ${I}_{N0}$ and ${A}_{(M0,N0)}$ are what we focus to screen out *N*_0_ and *M*_0_, meeting the constraints that the activation probability larger than 0.95 and *S*_APNF_ not less than 90% of on/off detection.

Constrained by the *S*_APNF_ not less than 90% of on/off detection, we set ${I}_{N0}\ge 0.9\times {\sum }_{k=1}^{+\infty }{E}_{k,t}$. In the main text, the activation probability is defined as the probability of detecting at least one event with more than *M* photons in *l* measurement cycles divided by the probability of detecting at least one event with more than *N* photons in *l* cycles. In experiment, the events with more than *N*_0_ photons have already been detected. Thus, we simplify the activation probability to:

$${P}_{A}=1-{\left(1-\frac{{I}_{M0}}{{I}_{N0}}\right)}^{{I}_{N0}}$$

which represents the probability of containing at least one event with more than *M*_0_ photons in ${I}_{N0}$. Considering the practicality and simplicity of the algorithm, we further convert the ${P}_{A}$ to:

$${P}_{A}=1-{\left(1-\frac{{I}_{M0}}{{I}_{N0}}\right)}^{l}\ge 0.95$$

where ${A}_{(M0,N0)}={I}_{M0}/{I}_{N0}$. Although it may result in some bias of ${P}_{A}$, it is beneficial for practical algorithm execution. Then, we can obtain ${A}_{(M0,N0)}\ge 1-{0.05}^{1/l}$. *N* and *M* are the maximized *N*_0_ and *M*_0_ while ensuring that ${I}_{N0}\ge 0.9\times {\sum }_{k=1}^{+\infty }{E}_{k,t}$ and ${A}_{(M0,N0)}\ge 1-{0.05}^{1/l}$, respectively. The ${E}_{k\ge M,{t}}$ in the 3D mask are used as references to filter ${E}_{k\ge N,{t}}$ in the processing pixel. This process can be regarded as pixelwise gating. In a particular situation when *M* = *N*, we use the events only in the surrounding pixels as references to filter the events in the processing pixel. The spatial distribution of *M* can be seen in Supplementary Information.

### Correcting the photon number distribution

The photon number distribution used to estimate the reflectivity in experiment is corrected by the matrix $\varPi$ corresponding to the POVM elements $\left\{{\pi }_{n}\right\}$ of the detector, which gives the probability ${\theta }_{i}^{(n)}$ of outcoming *n* when *i* photons are absorbed by the detector^35^. The matrix $\varPi$ are obtained though detector tomography, as described in the Supplementary Information, and is given by:

$$\varPi =\left[\begin{array}{ccccc}1 & 0 & 0 & 0 & 0\\ 0.0983 & 0.9017 & 0 & 0 & 0\\ 0 & 0.4270 & 0.5730 & 0 & 0\\ 0 & 0.1559 & 0.5634 & 0.2807 & 0\\ 0 & 0.0496 & 0.4109 & 0.4306 & 0.1088\end{array}\right]$$

A column of the matrix corresponds to the diagonal elements of the POVMs ${\pi }_{n}$. Here, we only consider the *n* no more than 4, since there are almost no events with more than 4 photons detected in the experiment. And we here extract the part where *i* is no more than 4 to make the matrix a square matrix. When the reflected signal has a photon number distribution *F* is detected by the detector, the output of the detector is expected to be:

$$P=l\times F\varPi$$

Both $F$ and $P$ are row vectors. And $P$ represents the photon number contents of detected events, $l$ is the number of repeated laser pulses. In experiment, $P$ is already known by recording the output of the detector. Then, we obtain the expected photon number contents $l\times F$ by:

$$l\times F={P\varPi }^{-1}$$

The corrected photon number contents are then used to estimate the reflectivity.

## Supplementary information

Supplementary Information for Noise tolerant LiDAR approaching the standard quantum-limited precision

## Data Availability

The data that support the findings of this study are available from the corresponding authors upon reasonable request.

## Code availability

The full code is available from the corresponding authors upon reasonable request.
