# Supplementary material for: Noise-tolerant LiDAR approaching the standard quantum-limited precision
Source: Light Sci Appl. 2025 Mar 26;14:138. doi: 10.1038/s41377-025-01790-5 (PMC11947159; doi:10.1038/s41377-025-01790-5)
Supplement: Supplementary file 1 — Supplementary Information for Noise tolerant LiDAR approaching the standard quantum-limited precision [file 41377_2025_1790_MOESM1_ESM.pdf]

Supplementary Information for

Noise-tolerant LiDAR approaching the standard quantum-limited precision

Haochen Li[1]†, Kaimin Zheng[2]†, Rui Ge[1,2]†, Labao Zhang[1,4]*, Lijian Zhang[2]*, Weiji He[3]*, Biao Zhang[1], Miao Wu[3], Ben Wang[2], Minghao Mi[2], Yanqiu Guan[1], Jingrou Tan[1], Hao Wang[1,4], Qi Chen[1], Xuecou Tu[1], Qingyuan Zhao[1], Xiaoqing Jia[1,4], Jian Chen[1], Lin Kang[1,4], Qian Chen[3] and Peiheng Wu[1,4]*

[1]Research Institute of Superconductor Electronics & Key Laboratory of Optoelectronic Devices and Systems with Extreme Performances of MOE, Nanjing University, Nanjing 210023, China
[2]College of Engineering and Applied Sciences, Nanjing University, Nanjing 210093, China
[3]Jiangsu Key Laboratory of Spectral Imaging and Intelligence Sense, Nanjing University of Science and Technology, Nanjing 210094, China
[4]Hefei National Laboratory, Hefei 230088, China
†These authors contributed equally to this work.
*Correspondence to E-mail: Lzhang@nju.edu.cn; Lijian.zhang@nju.edu.cn; hewj@mail.njust.edu.cn; phwu@nju.edu.cn.

Supplementary Note 1: Lidar equation

Lidar obtains the distance, morphology, reflectivity and other information of the target by transmitting brightness and a coherent laser and detecting the scattered photons. In addition to the detection ability of the detector, the most fundamental factor limiting the acquisition distance of the lidar is the intensity of the laser returned and received by the optical system. Therefore, it is necessary to establish an equation to describe the echo intensity. In the ideal case, the natural targets are Lambertian and have the same diffuse reflectance characteristics. Therefore, there is a negative correlation between the echo intensity and the square of the distance. The influence of atmospheric attenuation and optical systems should also be considered. Therefore, the lidar equation we established can be expressed as follows:

$$S\left(\mathrm{t}-\frac{2L}{c}\right)=\frac{FOV^2}{\theta_T^2}\frac{D^2 cos\theta_{\mathrm{target}}}{8R^2}\alpha\eta_{\mathrm{T}}\eta_{\mathrm{s}}\eta_{\mathrm{A}}^2 I\left(t-\frac{2L}{c}\right) \qquad (S1)$$

The distance between the target and the LiDAR is L, where θ_T is the diffusion angle of the laser and the FOV is the field of view of the receiver. The angle between the normal of the target surface and the laser optical axis is θ_{target}. The optical aperture of the receiver is D. η_A is the transmittance of the laser in air, which can be calculated by $\eta_A = \exp\left(-\int_0^L \sigma dr\right)$. σ is the atmospheric attenuation coefficient, which is closely related to the atmospheric visibility and laser wavelength. When atmospheric scattering is mainly considered the attenuation factor, the atmospheric attenuation factor can be simply calculated as $\sigma = \frac{3.91}{V}\left(\frac{\lambda}{550\,nm}\right)^{-q}$, where q is a parameter related to visibility V and λ is the wavelength of the laser. η_T is the transmittance of the laser in the optical system, and η_S is the coupling efficiency. α is the reflectivity of the target. *I(t-2 L/c)* represents the laser pulse, which has a temporal Gaussian distribution, while t is the flight time. The average signal photon number can be calculated by $\mu_p(t) = \frac{S(t-2L/c)\lambda}{hc}$, where h and c are the Planck constant and the speed of light in a vacuum, respectively.

Supplementary Note 2: The probability distribution of PNR detection

For photon-number-resolving (PNR) detection, the detected photon cluster consists of signal photons and noise photons. When assuming that signal photons and noise photons all follow a Poisson distribution, the distribution of mixed light can be regarded as the convolution of two Poisson distributions. The probability of detecting k photons can be expressed as follows:

$$\begin{aligned} p(k|t) &= \sum_{m=0}^{k} p_{\mathrm{p}}(m|t)p_{\mathrm{n}}(k-m) \\ &= \sum_{m=0}^{k} \frac{\mu_p^m(t)}{m!} e^{-\mu_{\mathrm{p}}} \frac{\mu_n^{k-m}}{(k-m)!} e^{-\mu_n} \\ &= \frac{e^{-(\mu_p+\mu_n)}}{k!} \sum_{m=0}^{k} \frac{k!}{m!\,(k-m)!} \mu_p^m \mu_n^{k-m} \end{aligned} \qquad (S2)$$

The second part on the right can be regarded as a binomial distribution of μ_{p} and μ_{n} and can be written as $(\mu_{\mathrm{p}} + \mu_{\mathrm{n}})^k$. Therefore, the probability can be expressed as:

$$p(k|t) = \frac{(\mu_p + \mu_n)^k}{k!} e^{-(\mu_p+\mu_n)} \qquad (S3)$$

The detection probability of the mixed light still follows a Poisson distribution with a mean signal photon of $\mu_{\mathrm{p}} + \mu_{\mathrm{n}}$.

Supplementary Note 3: SBR$_{APNF}$ versus mean signal photon number and measurement times

We show the map of SBR$_{APNF}$ versus the mean signal photon number μ_p and measurement time l for free running without a global gate (T_r = 50 μs) in Fig. S1(a). It can be seen that SBR$_{APNF}$ depends greatly on l and can still work when l is small. However, when μ_p and l are both small, it cannot work well since there are few events with multiple photons. In Fig. S1(b)&(c), we show the choices of N and M. It can be seen that N is 1 in most cases, while M varies with μ_p and l.

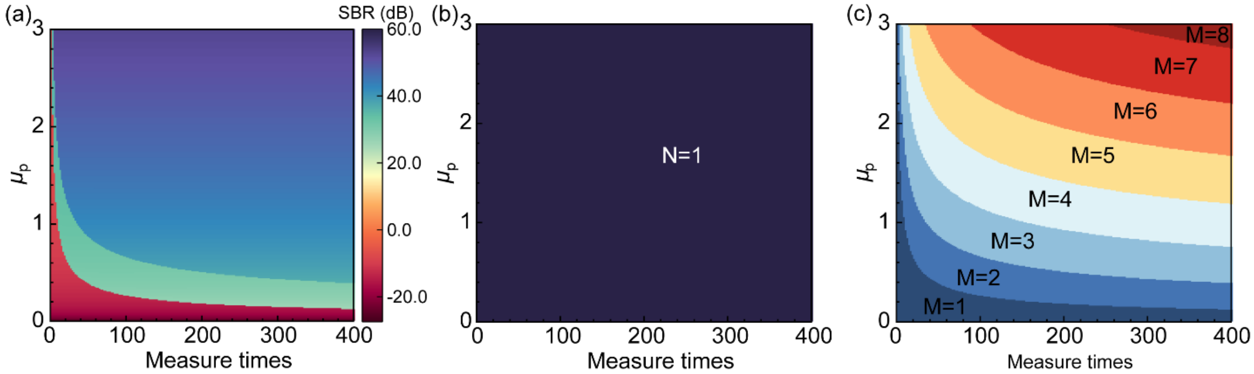

Fig. S1. SBR$_{APNF}$ as well as the relationship between SBR$_{APNF}$, M, N and l when the mean noise photon number is set as 10^{-4}. (a): The map of SBR$_{APNF}$ versus mean signal photon number μ_p and measurement times l. (b): The map of N versus μ_p and measurement times. (c): The map of M versus μ_p and measurement times.

Supplementary Note 4: SBR model of photon number threshold detection

When considering m photons detected in PNR detection, the corresponding SBR can be calculated as:

$$SBR_m = \frac{\sum_{i=1}^{f}[p(m|t) - p_p(0|t)p_n(m)]_{t=i*\Delta t}}{p_n(m)_{\Delta t}f} \qquad (S4)$$

To simplify the formula, we introduce $p_s(m|t) = p(m|t) - p_{\mathrm{p}}(0|t)p_{\mathrm{n}}(m)$. Since the mean photon number of noise is usually less than that of the signal, the $\mathrm{SBR_m}$ with a larger m is also larger. If we apply a photon number threshold to discard events with fewer than N photons, the $\mathrm{SBR_{THD}}$ can be expressed as:

$$\mathrm{SBR_{THD}} = \frac{\sum_{k=N}^{M}\sum_{i=1}^{f}[p_s(k|t)]_{t=i*\Delta t}}{\sum_{k=N}^{M} p_{\mathrm{n}}(k)_{\Delta t}f} \qquad (S5)$$

In Fig. S2, we show $\mathrm{SBR_{THD}}$ with different photon number thresholds for free running without a global gate (T_{r} = 50 μs). As shown in Fig. S2(a), with the improvement in N, the $\mathrm{SBR_{THD}}$ improves considerably. To make a comparison between $\mathrm{SBR_{APNF}}$, we also show the map of $\mathrm{SBR_{THD}}$ versus μ_{p} and l when N is set to 2. Although simply abandoning events with fewer photons can greatly improve SBR, it may cause the loss of signal. We discuss the topic in Supplementary Note 5.

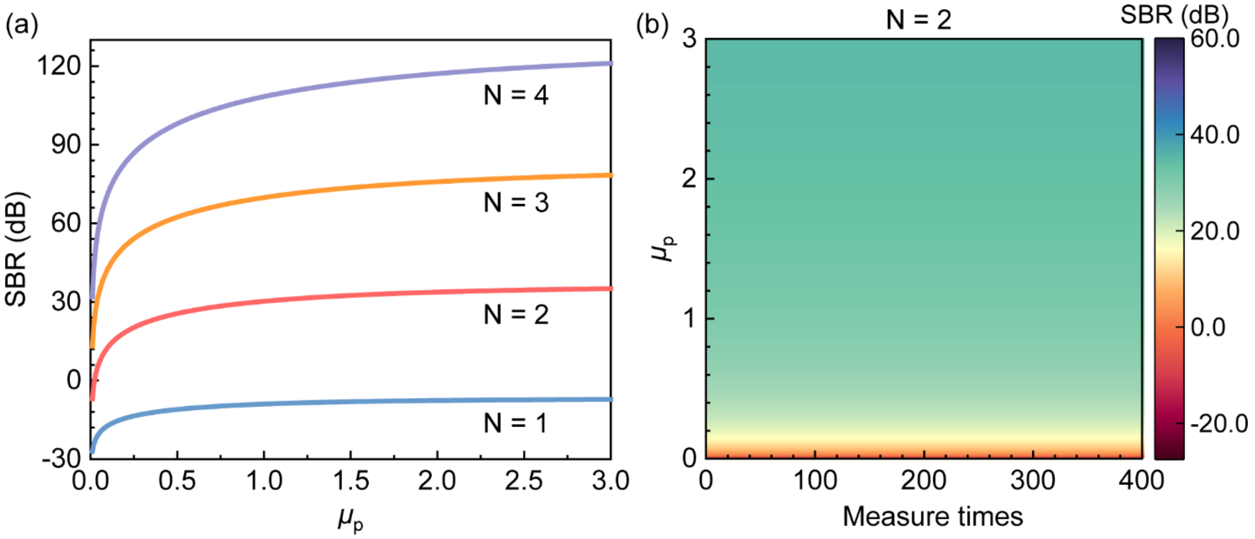

Fig. S2. The value of $\mathrm{SBR_{THD}}$. (a): $\mathrm{SBR_{THD}}$ versus μ_{p} with different N. (b): The map of $\mathrm{SBR_{THD}}$ versus μ_{p} and measurement times with N=2. This image is used for comparison with Fig. S1(a).

Supplementary Note 5: The signal intensity with different methods

We calculate the overall signal intensity of different detection methods, which is defined as the overall probability of detecting a signal event in a unique measurement, as shown in Fig. S3. Since on/off detection detects all events, its signal intensity is the largest and varies with μ_p. Compared with on/off detection, simply abandoning events with one photon in PNR detection obviously reduces the signal intensity. In contrast, APNF does not reduce the signal intensity significantly.

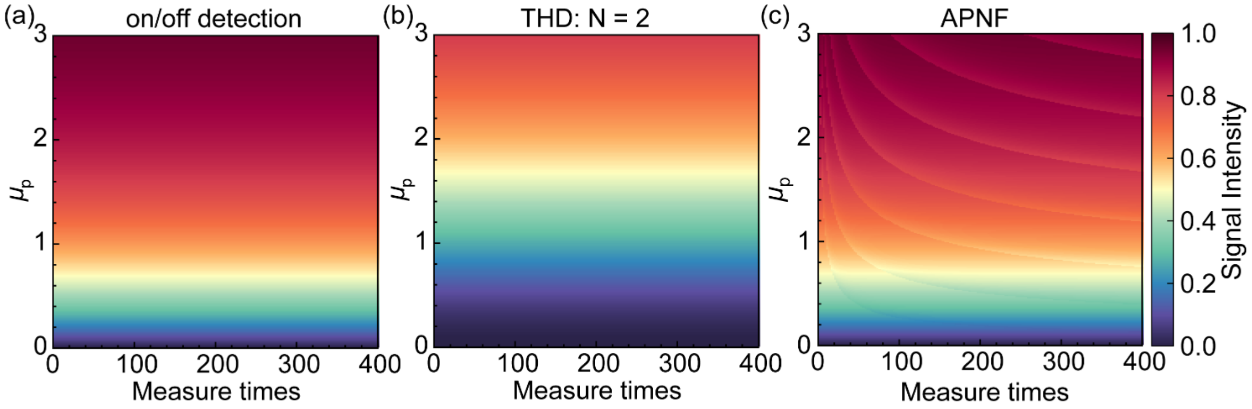

Fig. S3. The maps of signal intensity with different methods. The signal intensity is defined as the probability of detecting a signal in a unique measurement. (a) Map of signal intensity with on/off detection. (b) Map of signal intensity when setting the photon number threshold as 2 in threshold detection. (c) The map of signal intensity after APNF.

Simple threshold detection with a discrimination threshold that is too high may cause incompleteness of the generated image. The application of APNF can avoid this problem, as shown in Fig. S4. In Fig. S4(a), we show the detailed 3D point cloud of the pylon when setting the photon number threshold as 2 in threshold detection, and the number of measurements is 20. It can be seen that some details of the pylon are lost. In contrast, the 3D point cloud with APNF is more complete, as shown in Fig. S4(b). Similarly, the quality of the reconstructed image with APNF is better, as shown in Fig. S5.

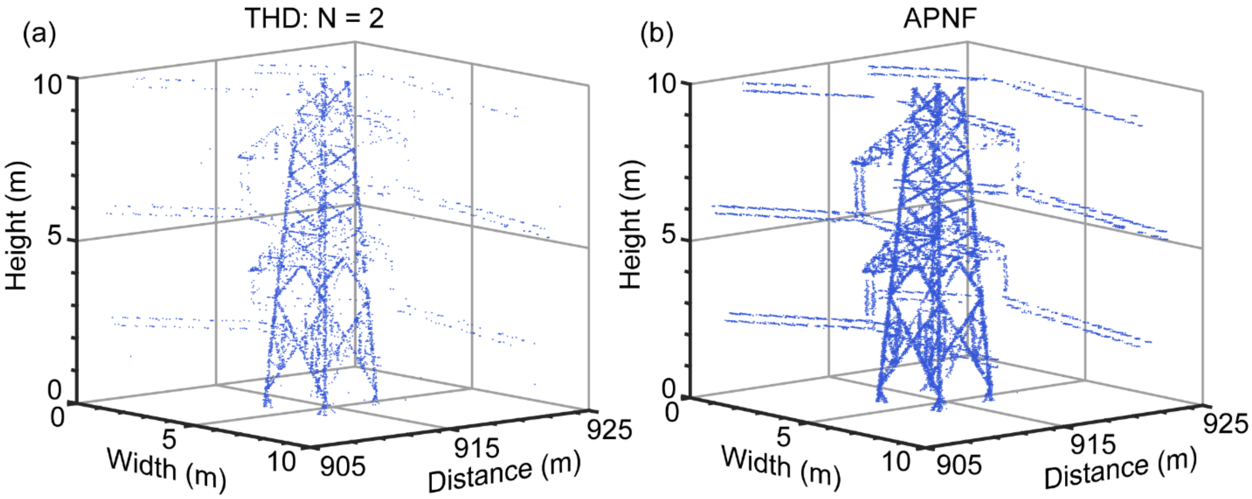

Fig. S4. The 3D point clouds with different methods when the number of measurements is 20. (a) The 3D point cloud when setting the photon number threshold as 2 in threshold detection. (b) The 3D point cloud with APNF.

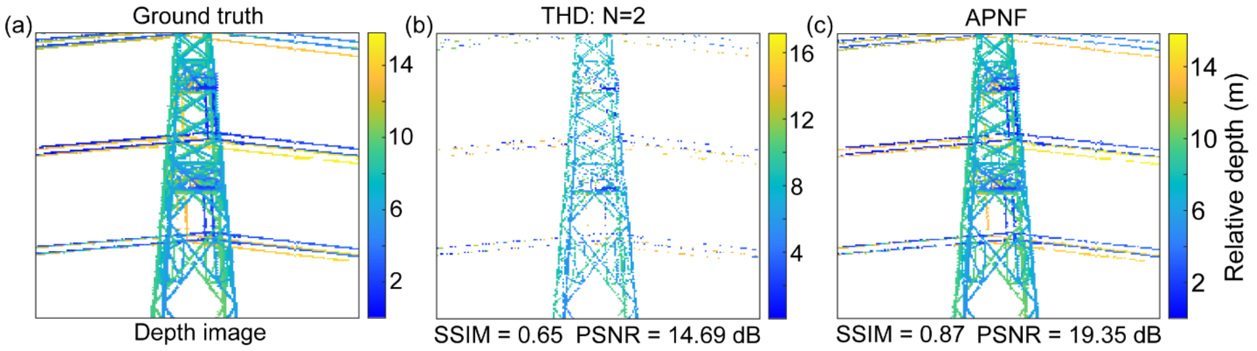

Fig. S5. Comparison between the reconstructed depth images. (a) The ground truth obtained through long-term detection (also shown as Fig. 5(a) in the main text). (b) The depth image reconstructed from the 3D point cloud shown in Fig. S4(a). (c) The depth image reconstructed from the 3D point cloud shown in Fig. S4(b) (also shown as Fig. 5(c) in the main text).

Supplementary Note 6: ASE noise

In the experiment, since we use a laser source with an optical amplifier and coaxial optical system, the amplifier spontaneous emission (ASE) noise inside the laser can easily enter the detector through backscattering, as shown in Fig. S6(a) and (b). The introduction of ASE noise interferes with the detection and reduces the signal background ratio (SBR) of the final result. In previous work, some researchers proposed using an acousto-optic modulator to block ASE noise and achieved excellent results [1]. In the experiment, we found that the influence of ASE noise can be ignored in multiphoton detection, as shown in Fig. S6(c) and (d). Therefore, it is possible to improve SBR by using multiphoton information.

As the ASE noise follows a Poisson distribution, it can be linearly superimposed with the signal, so it does not affect the estimation of the reflectivity.

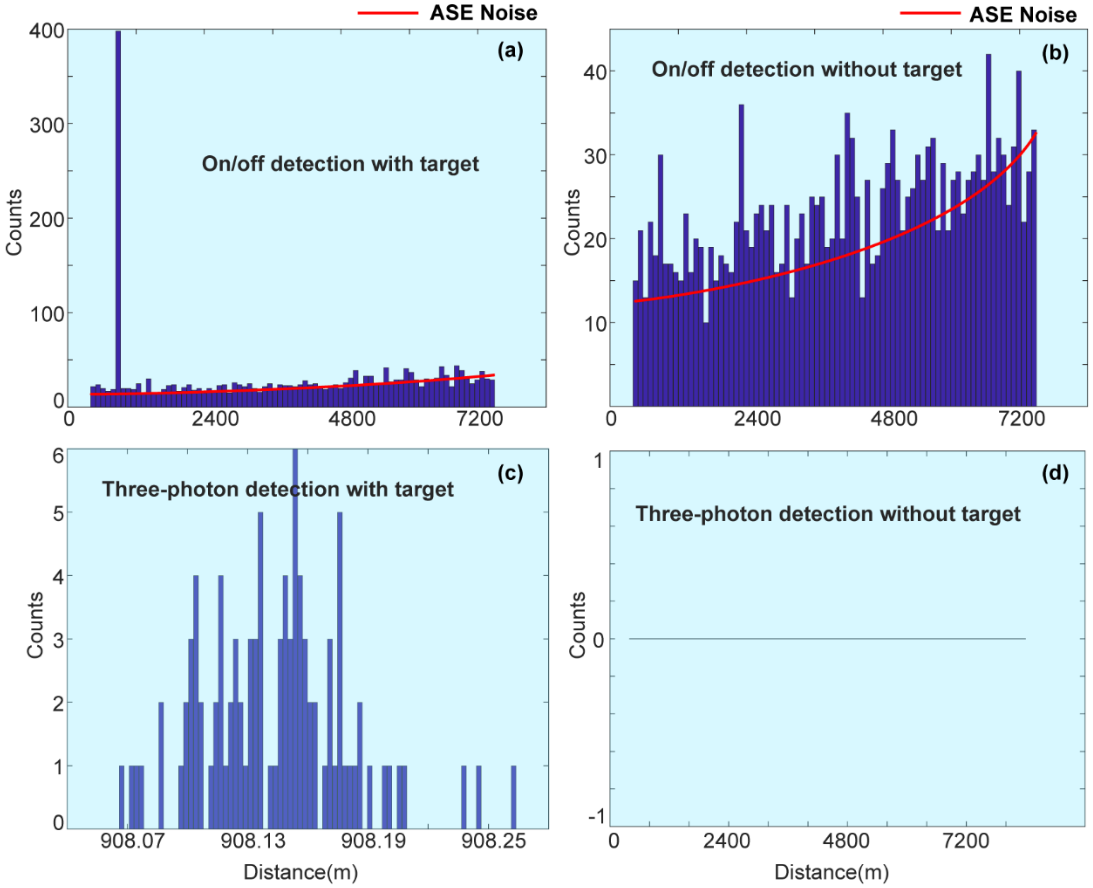

Fig. S6. The ASE noise in the experimental results. (a)-(b): Histogram of on/off detection data. (c)-(d): Histogram of three-photon detection data.

Supplementary Note 7: Improvement in the dynamic range with PNR detection

Compared with the standard quantum limit (SQL, indicated by quantum Fisher information), Fig. S7 shows the attenuation of Fisher information with detectors that can resolve up to 1, 4, 8, 12 and 16 photons. Taking the point 3 dB lower than the SQL as a reference, the dynamic range of AT_{16} is 11.87 dB larger than that of AT_1, which means that photon-number-resolving detection has a larger dynamic range than on/off detection.

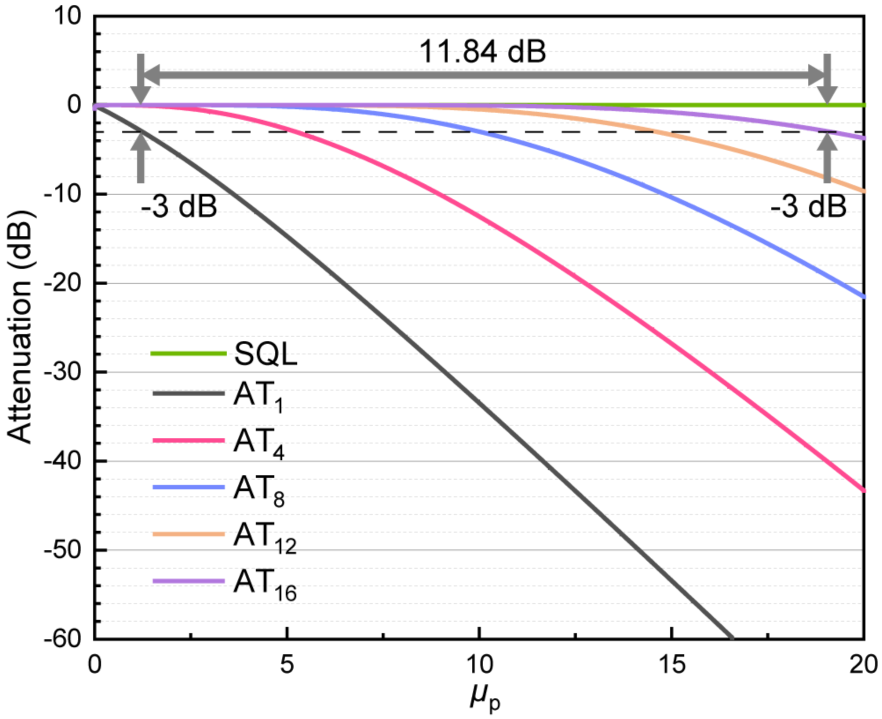

Fig. S7. Attenuation of Fisher information compared with the standard quantum limit when assuming different maximum resolvable photon numbers. AT_1, AT_4, AT_8, AT_{12} and AT_{16} represent attenuation with detectors that can resolve up to 1, 4, 8, 12 and 16 photons, respectively.

Supplementary Note 8: Fisher information of photon number threshold detection

For photon-number threshold detection, the detection probability model can be expressed as:

$$p_{\mathrm{THD}}(k|t) = \begin{cases} \sum_{m=0}^{N-1} p(m) & k = 0 \\ 1 - \sum_{m=0}^{N-1} p(m) & k = 1 \end{cases} \quad (S6)$$

Thus, we can calculate the Fisher information according to formula (1) in the manuscript. The results are displayed in Fig. S8. In this figure, F_1 to F_{16} represent Fisher information using one-photon to 16-photon threshold detection. $F_{\max}$ represents Fisher information when using the best threshold under any condition. Compared with PNR detection (shown in Fig. 2(b) in the manuscript), the Fisher information of photon-number threshold detection can only approach quantum Fisher information (SQL) in a short range for each threshold. If we use the optimum threshold at any time, we can reach the max Fisher information ($F_{\max}$), which is still 1.91 dB lower than the QFI when μ_{p} is 10.

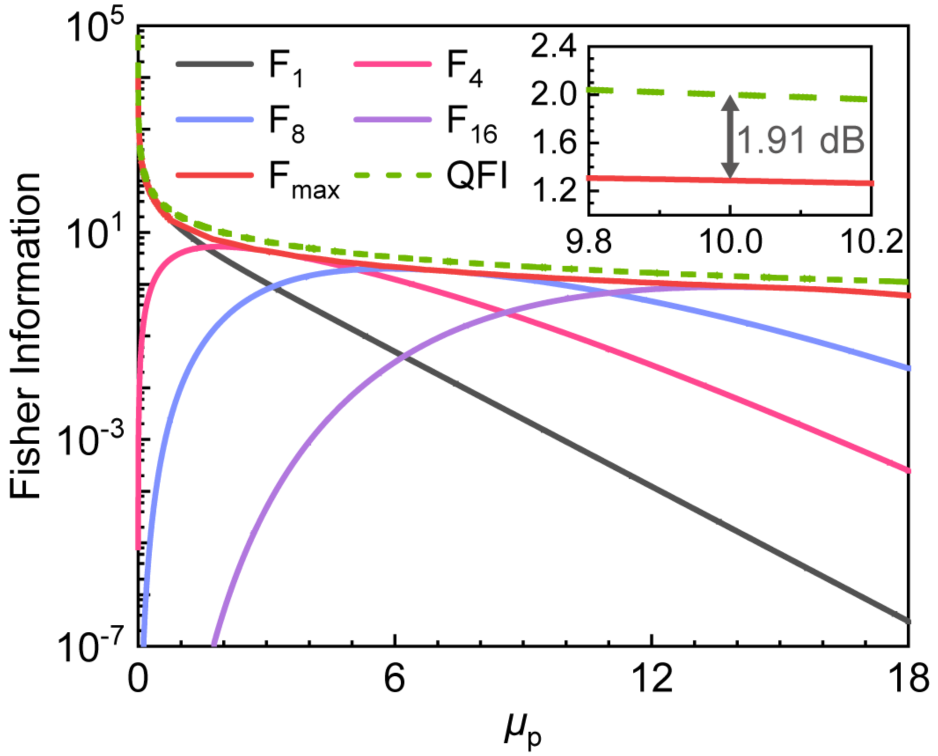

Fig. S8. Fisher information of different photon number threshold detections. F_1 to F_{16} represent Fisher information using one-photon to 16-photon threshold detection. $F_{\max}$ represents Fisher information when using the best threshold under any condition.

Supplementary Note 9: Simulated reflectivity estimation

As we mentioned in the manuscript, we use a grayscale image to test the reflectivity estimation capability of the proposed method. The original image and the image with superimposed Gaussian noise can be seen in Fig. S9. In fact, we use the image with superimposed Gaussian noise to generate detected events.

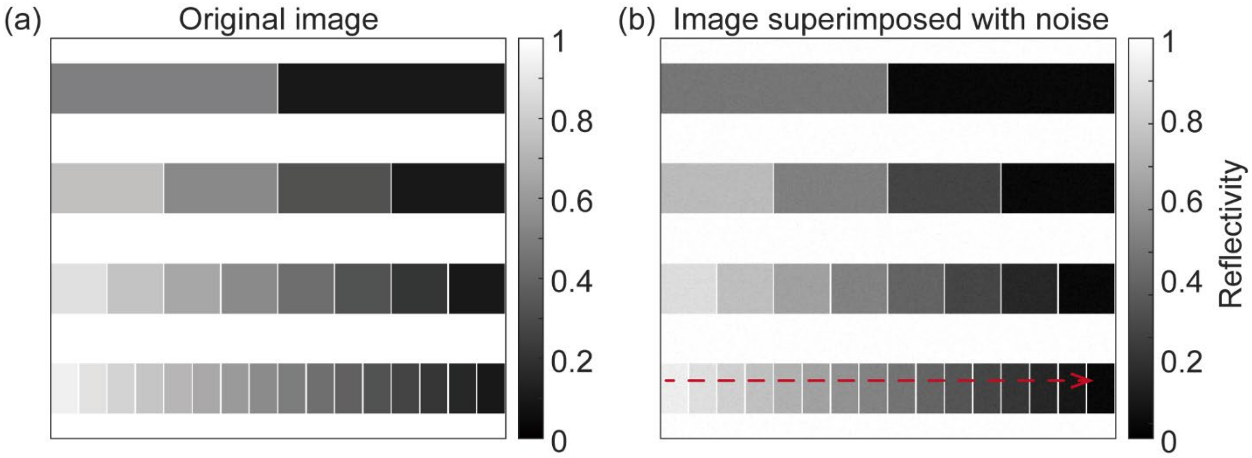

Fig. S9. (a) The original gray image. (b) The gray image superimposed with Gaussian noise.

Figure. S10 shows the results of simulated reflectivity estimation with PNR detection, which can resolve up to 16 photons and on/off detection. 10 mpp means taking 10 measurements per pixel. Although the results of PNR detection are greatly disturbed by noise due to limited measurement times, it can still resolve 16 reflectivity levels. The white area in the results of on/off detection represents an infinite value, which indicates saturation and failure in estimating the mean signal photon number. Figure. S11 shows the estimated mean signal photon number along the red dashed arrow shown in Fig. S9(b). The blue lines show the average value of 15 rows.

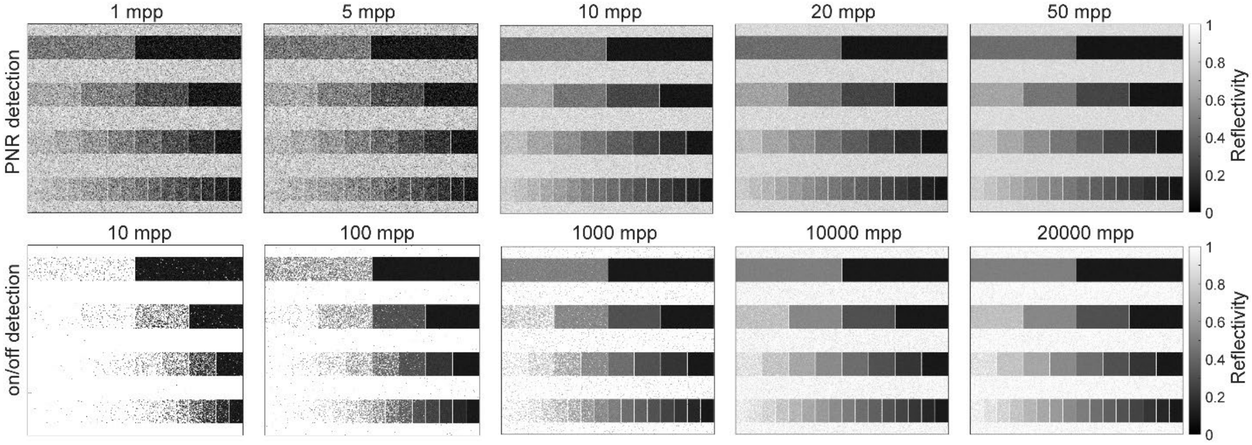

Fig. S10. Simulated results of reflectivity estimation with PNR detection, which can resolve up to 16 photons and on/off detection. 10 mpp means taking 10 measurements per pixel.

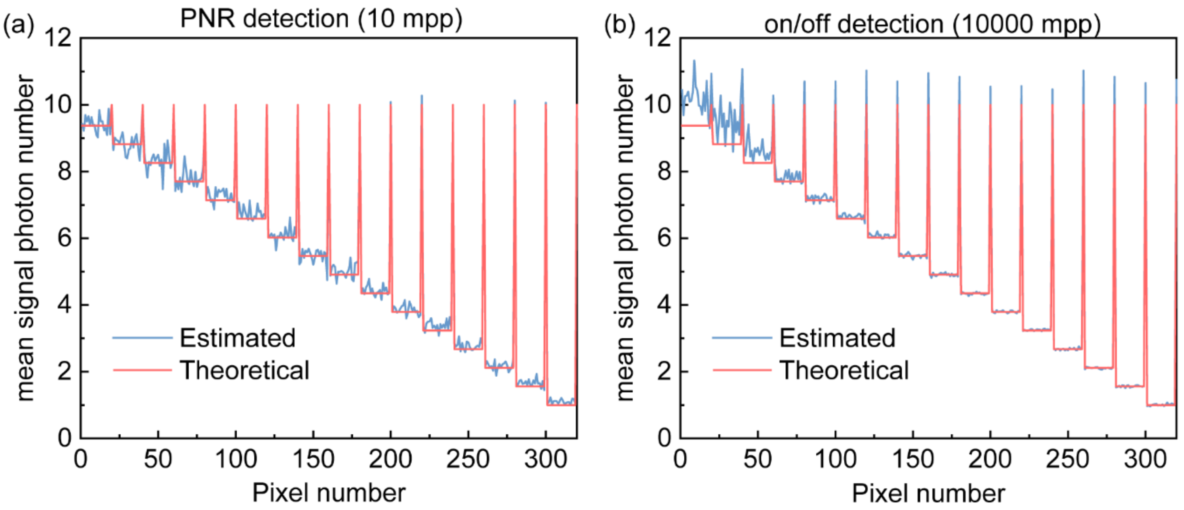

Fig. S11. The estimated and theoretical mean signal photon number along the red dashed arrow is shown in Fig. S8(b).

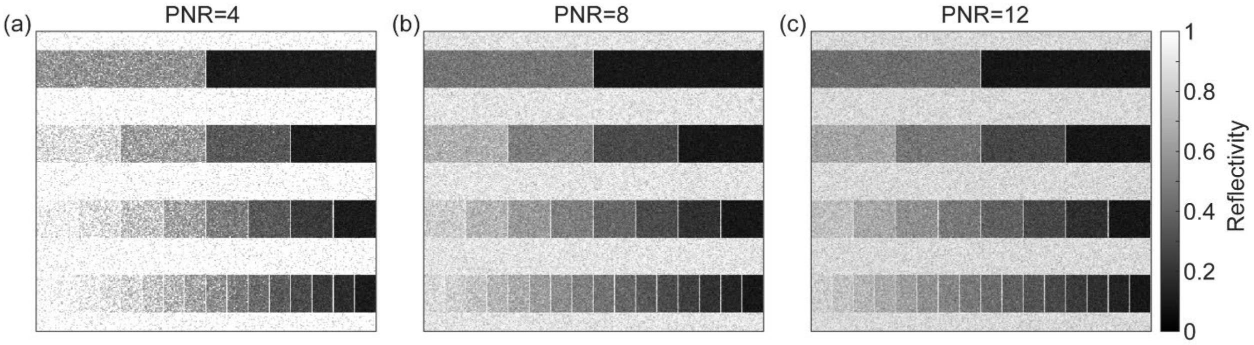

Fig. S12. Simulated results with different PNR capabilities based on 10 measurements per pixel.

Supplementary Note 10: Shannon Information model

The accuracy we measuring μ_p can be convert into the probability we distinguishing between two adjacent reflectivities. To quantify the probability, we here introduce a parameter named statistical distance [2], which can be calculated as:

$$d = \sqrt{l}\frac{\mu_{step}}{2\sigma_0} \quad (S6)$$

where l is the measurement times, $\mu_{step}=\mu_{p\_max}/R$ is the difference between reflected signal of two adjacent reflectivity, and μ_{p_max} is the maximized μ_p when reflectivity is unit, R is the number of reflectivity levels. While σ_0 is the standard deviation taking only one measurement. As we have known the Fisher Information about μ_p, the σ_0 can be calculated as $\sigma_0 = \sqrt{1/F(\mu_p)}$ according to the Cramer-Rao bound. Using the Gauss error function, the probability we discriminate two adjacent reflectivity can be expressed as:

$$P(d) = \frac{1 + \text{erf}\left[d(\mu_p)/\sqrt{2}\right]}{2} \quad (S7)$$

Here, μ_p is the echo photon number of the larger reflectivity. When d is 0, then P=0.5, which means that the measurement cannot offer us any information helps to the discrimination. To weigh the amount of information, we introduce the Shannon Information (I):

$$I_s(d) = log_2(P) - log_2\left(\frac{1}{2}\right) \quad (S8)$$

When d=0 and P=0.5, the Shannon Information is 0 bit, representing no information obtained. When P=1, the Shannon Information is 1 bit, which indicates that we can completely distinguish the two reflectivities. If there are R reflectivities need to be distinguished, the total Shannon Information can be calculated as:

$$I(\mu_{\text{step}}, R, l) = \sum_{i=1}^{N} log_2\left\{1 + \text{erf}\left[\sqrt{l}\frac{\mu_{step}\sqrt{F(i\mu_{step})}}{2\sqrt{2}}\right]\right\} \quad (S9)$$

In Fig. S13(a), we provide the results with different max-resolvable photon numbers when μ_{p_max}=10 and there are 16 reflectivity levels. I_1, I_4, I_8, I_{12} and I_{16} represent the Shannon Information with detections that can resolve up to 1, 4, 8, 12 and 16 photons, respectively. And I_Q is the I calculated from the quantum Fisher Information, which is determined by the shot-noise limit (standard quantum limit). It can be seen that I_Q costs the lest number of measurements to reach 16 bits, which indicates we can fully resolve the 16 reflectivities. And the curve of I_{16} almost coincides with I_Q, showing the ability to approach the standard quantum limit. Here, we define the point when d=1 as the point we can just right distinguish two reflectivities. In this case, the P is about 0.84 and the I_s is about 0.75 bits. Since d decrease with μ_p according to the trend of FI versus μ_p, we can just right distinguish N reflectivities as long as $d(\mu_{p\_max})$=1. In Fig. S13(a), the black arrows mark the number of measurements needed to satisfy $d(\mu_{p\_max})$=1. As a result, 104 measurements are needs for PNR detection with 16 photons resolvable and about 227800 measurements are needed for on/off detection. And there is about 33.4 dB enhancement using PNR detection, which

is consistent with the Fisher information. When μ_{p_max} is 1, these values are 1035, 1788 and 2.4 dB, respectively.

In Fig. S13(c) and (d), we show the simulated reflectivity reconstruction with 10 mpp for PNR=16 detection and 20000 mpp for on/off detection when μ_{p_max}=10, respectively. We can just right resolve the 16 reflectivities (marked by red dashed box) through visual observation in this case. The values are about 1 tenth of the Shannon Information prediction. This is because the eyes do not focus only on one pixel but also its surrounding pixel to extract the feature of target when observing. According to Johnson Criteria in thermal imaging [3], more than 10 pixels along the critical dimension are needed to achieve the identification. Observing multiple pixels simultaneously is equivalent to increasing the actual number of measurements. Thus, less measurements per pixel are required. Extended Data Fig. 3 shows the detailed estimation results along the blue dashed arrow shown in Extended Data Fig. 2(a). And Fig. S14(a) provides the results of a single row when taking 10 mpp. It can be seen that 10 mpp fails to divide different reflectivities, while 100 mpp are successful. Besides, the mean value of 12 rows is consistent with the results of 100 mpp, as shown in Fig. S14(b). Both of them can just right show 16 reflectivity levels.

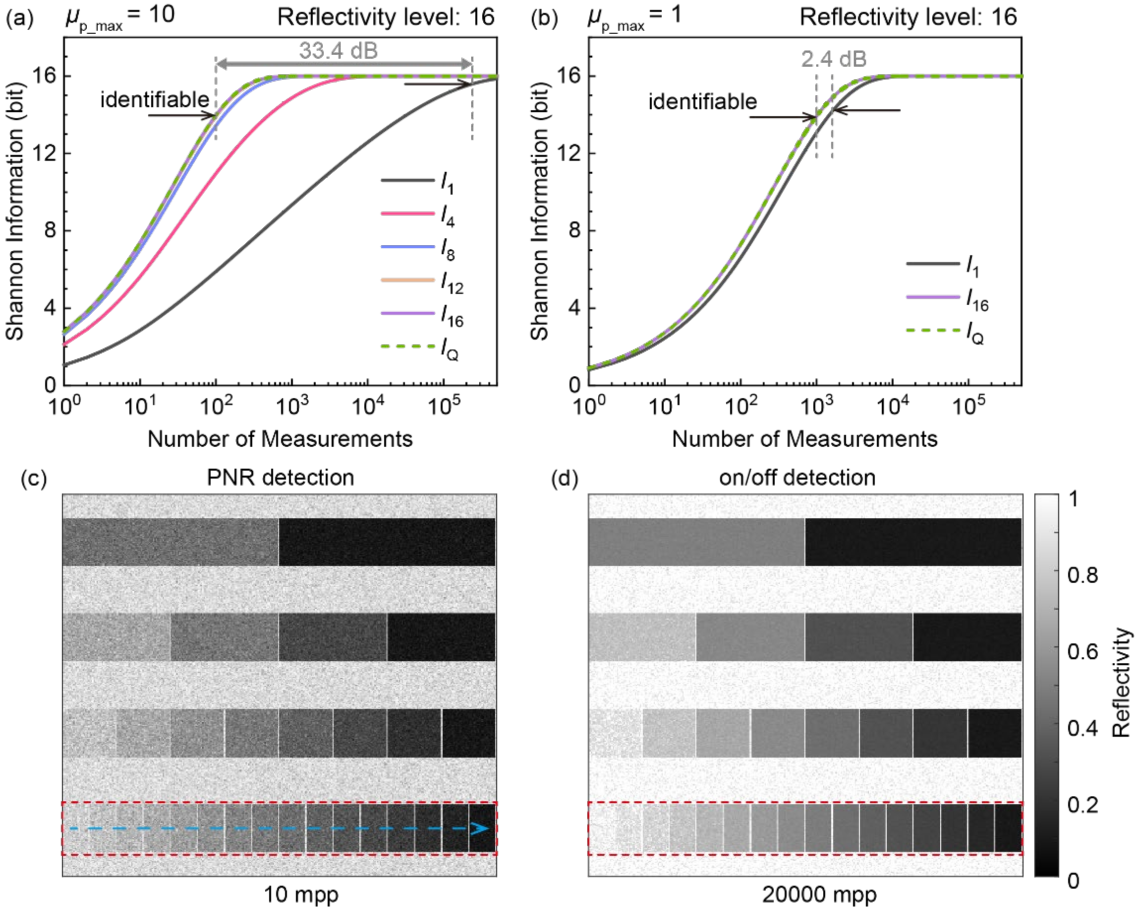

Fig. S13. Shannon Information and simulated reflectivity reconstruction when there are 16 reflectivity levels. (a) The Shannon information when μ_{p_max}=10. I_1, I_4, I_8, I_{12} and I_{16} represent the Shannon Information with detections that can resolve up to 1, 4, 8, 12 and 16 photons, respectively. And I_Q is the I calculated

from the quantum Fisher Information. The black arrows mark the points when statistical distance d=1, at which we can just right resolve 16 reflectivities. (b) The Shannon information when μ_{p_max}=1. (c) The reconstructed reflectivity image taking 10 measurements per pixel (mpp) with PNR detection which can resolve up to 16 photons when μ_{p_max}=10. (d) The reconstructed reflectivity image taking 20000 mpp with on/off detection when μ_{p_max}=10.

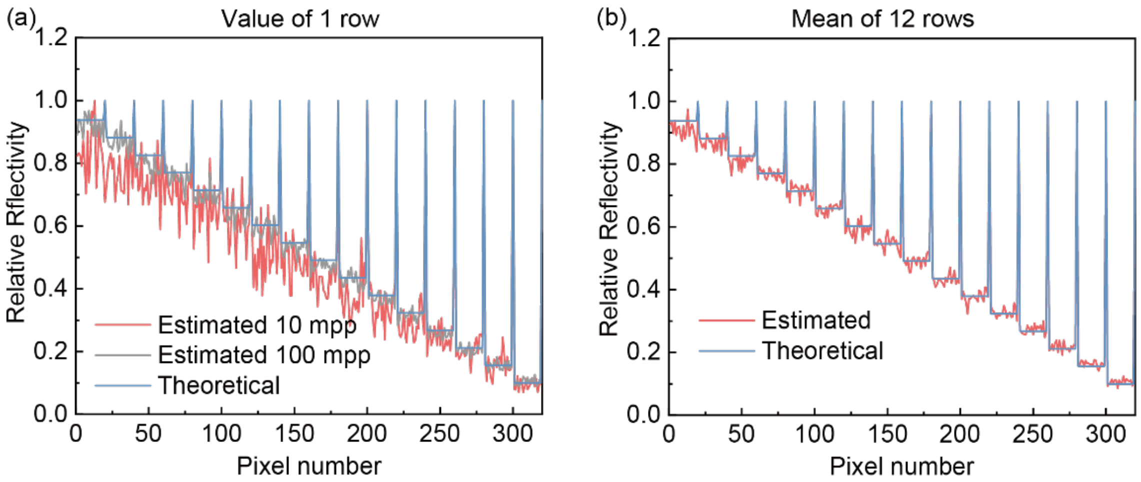

Fig. S14. The detailed estimation results along the blue dashed arrow shown in Fig. S13(c). (a) The results of a single row. The red line gives the value of 10 mpp, and the blue line represents the theoretical results. The gray line gives the results when taking 100 mpp, which can just right divide 16 reflectivity levels. (b) The mean value of 12 rows when taking 10 mpp. The results are consistent with the gray line shown in (a).

We then analysis the relationship between the Shannon Information and number of reflectivity levels when taking a given detection method and a fixed μ_{p_max}. Figure. S15 show the normalized Shannon Information versus measurement times under different reflectivity levels when taking PNR=16 detection and assuming μ_{p_max}=10. It can be seen that the number of measurements required to achieve the unity normalized Shannon Information increases with the reflectivity levels, as the black dashed arrow indicates. This trend is in line with the common intuition that more measurements are needed to realize higher accuracy or resolution.

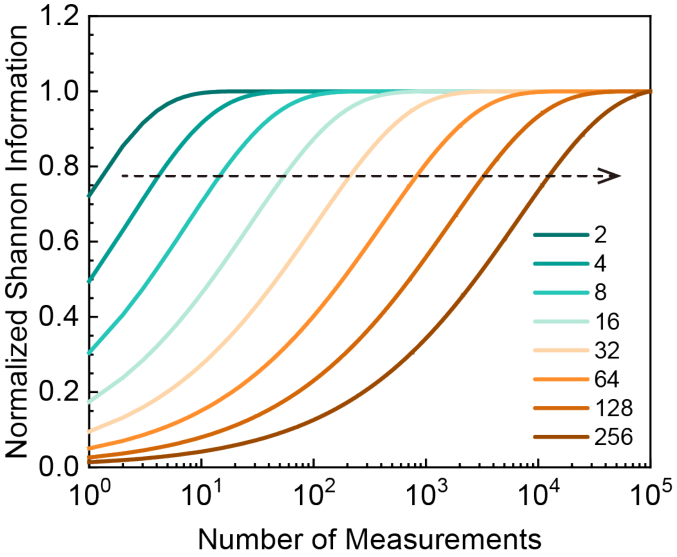

Fig. S15. The normalized Shannon Information versus the number of measurements under different reflectivity levels when taking PNR=16 detection and assuming μ_{p_max}=10. The black dashed arrow indicates that the number of measurements required to achieve the unity normalized Shannon Information increases with the reflectivity levels.

Similarly, we also analysis the relationship between the Shannon Information versus the number of measurements assuming different μ_{p_max} when there are 16 reflectivity levels and taking PNR=16 detection, as shown in Fig. S16. The number in the Fig. S16(a) represents the value of μ_{p_max}. It can be seen that with the increase of μ_{p_max}, the number of measurements required to reach the saturated Shannon Information also increase. Equally, the case with a larger μ_{p_max} has a larger Shannon Information when taking the same number of measurements, which indicates that the accuracy or resolution of measurements increase with the signal intensity.

In Fig. S16(b), we provide the curve of min measurement times required to just right resolve 16 reflectivities versus the μ_{p_max} with PNR=16 detection. The min measurement times are also defined as the value which make $d(\mu_{p\_max})$=1. And the red dashed curve in the Fig. S16(b) represents the results limited by the shot-noise of photons. Clearly, the red dashed line drops with the increase of μ_{p_max}. The blue line (the results of PNR=16 detection) is coincident with the shot-noise limit at beginning and then bends upwards with the μ_{p_max} increase. The blue curve finally rise with the increase of μ_{p_max} after the equilibrium point (14.7, marked by the black arrow). It is caused by the loss of photon number information due to the failure of the detector to resolve photon number larger than 16. And at the equilibrium point, the min measurement times is 82. We also provide the results using on/off detection in Fig. S17. Different from the results using PNR detection, it coincides with the shot-noise limit within a small range less than 1 and rapidly increase after the equilibrium point (1.6). And the min measurement times at the equilibrium point is 1601. These results prove the enhancement of PNR detection on LiDAR, especially approaching the quantum-limited precision in reflectivity measurement.

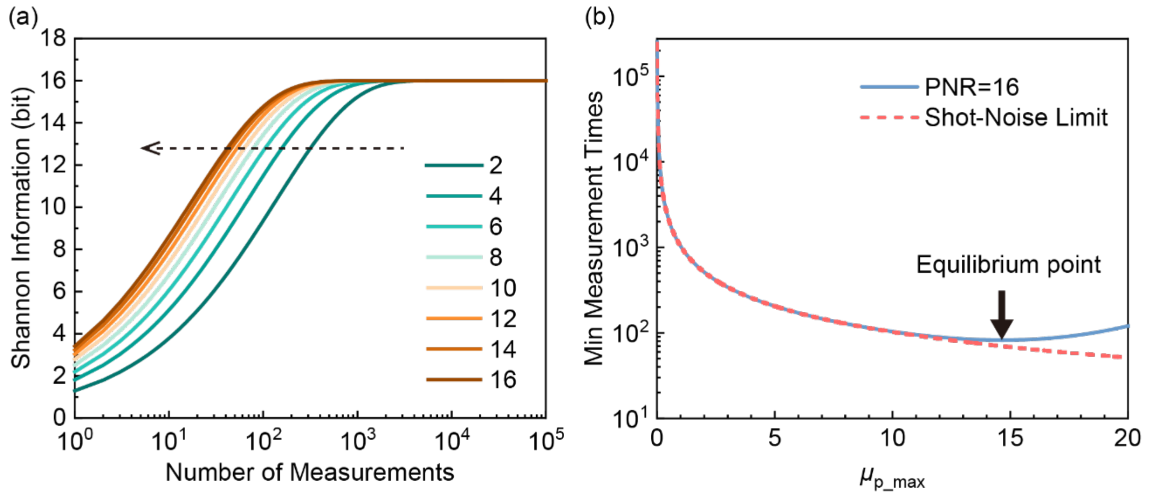

Fig. S16. The Shannon Information and the min measurement times required to identify 16 reflectivities using PNR detection which can resolve up to 16 photons. (a) The Shannon Information versus the number of measurements with different μ_{p_max}. The numbers in the graph represent the μ_{p_max}. The black dashed arrow marks the trend of the measurement number to obtain the same Shannon Information with the increase of μ_{p_max}. (b) The min measurement times required to identify 16 reflectivities versus μ_{p_max}. The red dashed line the results limited by the shot-noise of photons, and the blue line is the results obtained using PNR detection. The black arrow marks the equilibrium point between the descending and rising of the curve.

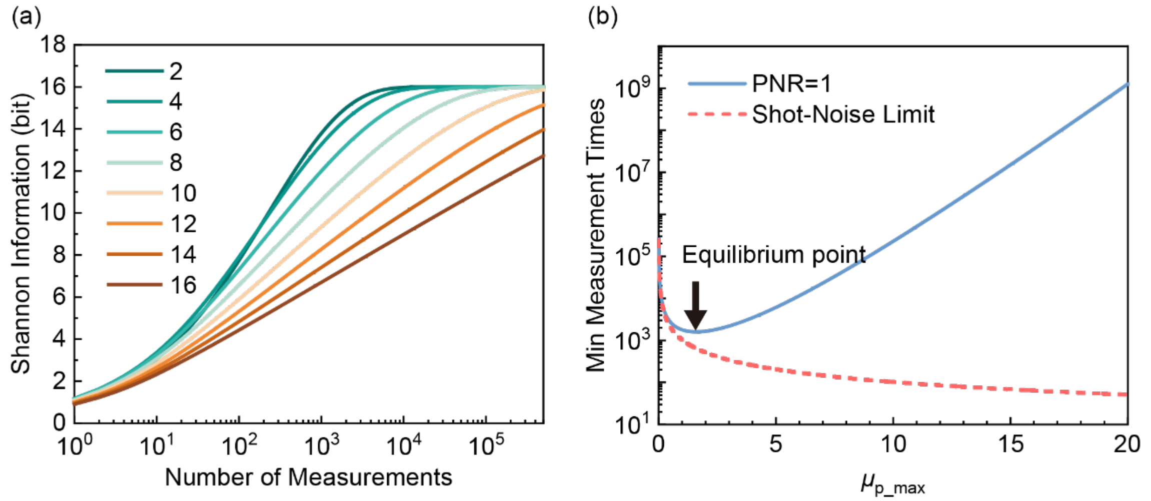

Fig. S17. The Shannon Information and the min measurement times required to identify 16 reflectivities using on/off detection. (a) The Shannon Information versus the number of measurements with different μ_{p_max}. (b) The min measurement times required to identify 16 reflectivities versus μ_{p_max}.

Furthermore, we mapped the min measure times versus the reflectivity level and the μ_{p_max} based on the above theoretical model, as shown in Fig. S18. And Fig. S18(a), (b) and (c) show the map of on/off detection, PNR=16 detection and the shot-noise limit, respectively. The purple dash-dot

line in the maps are contour lines. Generally, less measurements are required to reach the same reflectivity resolution with the increase of signal intensity (μ_{p_max}) before the equilibrium line for both on/off detection and PNR detection. And more measurements are required to reach a higher reflectivity resolution for a fixed signal intensity. Both of these results are in line with the intuition. However, the PNR detection has a larger dynamic range than on/off detection, and nearly always cost less measurements to realize the same reflectivity resolution. Moreover, the map of PNR detection is similar with that of the shot-noise limit, which indicates the quantum-limited performance of PNR detection.

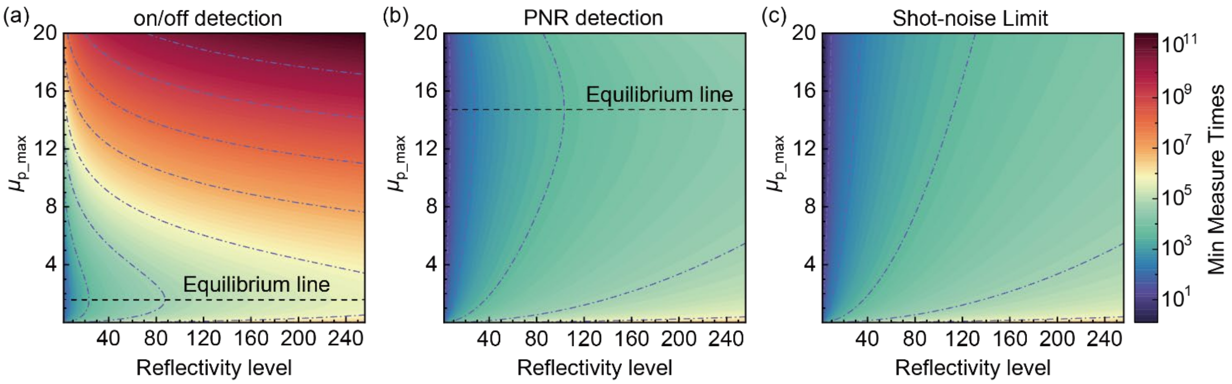

Fig. S18. The map of identification requiring min measure times versus μ_{p_max} and reflectivity level. The purple dash-dot lines in the maps are the contour lines. (a) The map of on/off detection. The black dashed line is the equilibrium line which marks the transition of the min measure times from decrease to increase with μ_{p_max}. (b) The map of PNR detection which can resolve up to 16 photons. (c) The map of the shot-noise limit.

Then we compare the performance of PNR=16 detection and on/off detection through dividing the min measure times of on/off detection by that of PNR detection. The result is shown in Fig. S19. It can be seen that PNR detection always takes less measurements than on/off detection to resolve the same reflectivity levels. But when μ_{p_max} is small, the gain of PNR detection is also small. For example, when μ_{p_max} is 0.44, the gain is about 1 dB. Moreover, the gain only depends on the μ_{p_max}, in consistent with the Fisher information.

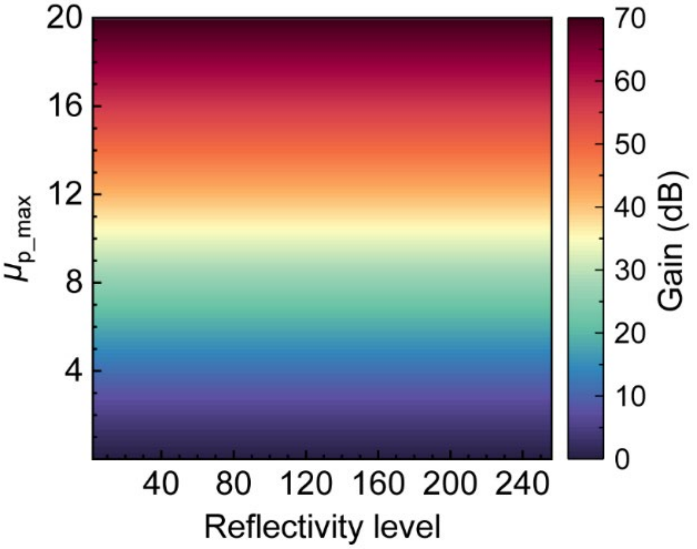

Fig. S19. The gain of PNR=16 detection compared to on/off detection in the reflectivity resolution. The gain is defined as $10\log_{10}(*)$ of the min measure times of on/off detection divided by that of PNR detection.

In conclusion, based on the Fisher Information analysis, we establish a Shannon Information model to analyze the advantages brought by PNR detection and prove its approaching quantum-limited performance in a wider dynamic range than on/off detection. This model clearly shows how improving Fisher Information helps to improve the sensitivity of LiDAR measurement and correctly predict the performance of LiDAR in reflectivity imaging under different situations, which can help extensive researchers to design LiDAR and implement single-photon computational imaging experiments.

Supplementary Note 11: Experimental intensity estimation

Figure. S20 gives images of the estimated mean signal photon number $\hat{\mu}_{\mathrm{p}}$ obtained by Bayesian estimation with different PNRs. PNR = 1 to PNR = 4 indicates that the detector can resolve up to 1 to 4 photons. The height and color represent the value of the mean signal photon number ($\hat{\mu}_{\mathrm{p}}$). The standard deviation of the estimation corresponds to Fig. 3(e)-(h) in the manuscript.

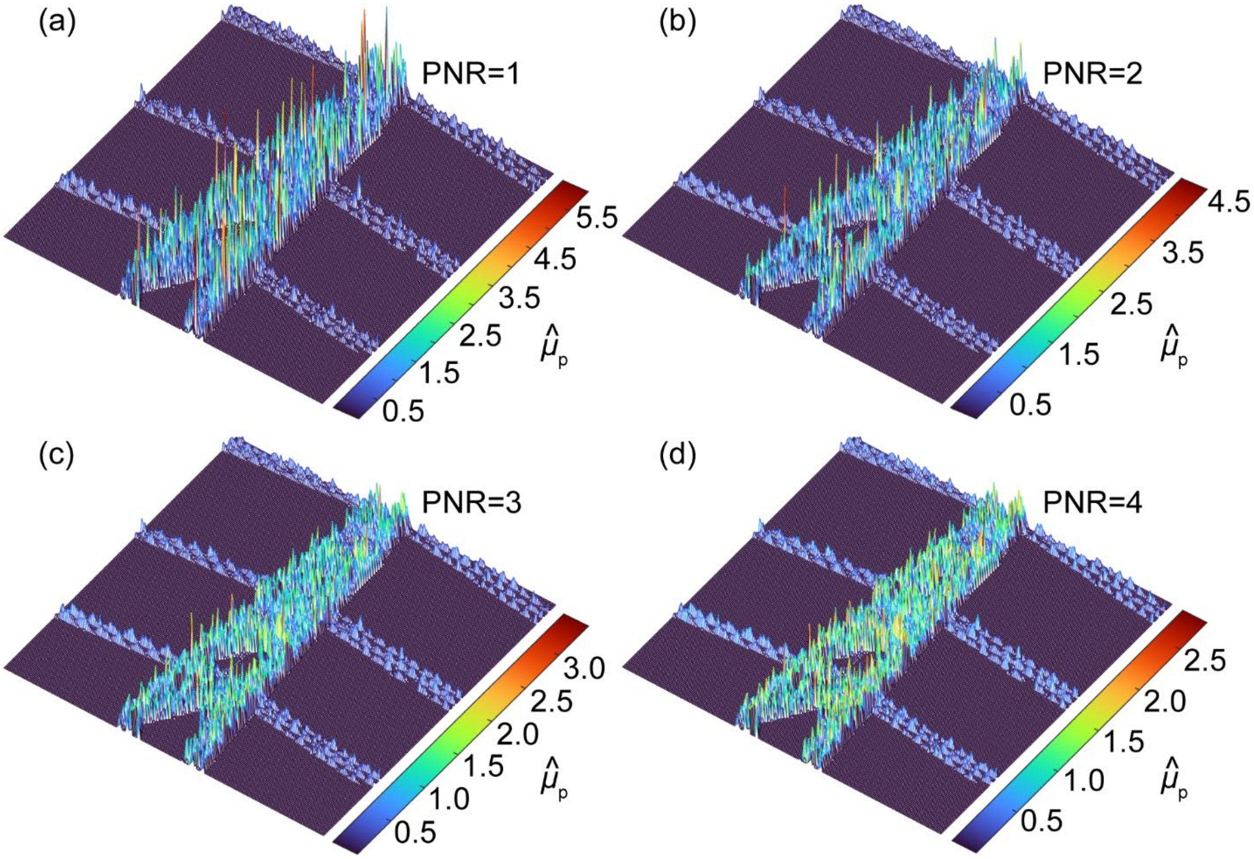

Fig. S20. Results of intensity estimation. PNR = 1 to PNR = 4 represent that the detector can resolve up to 1 to 4 photons. The height and color represent the value of the mean signal photon number (μ_{p}).

Supplementary Note 12: Details of theoretical calculation of SBR$_{APNF}$

First, assuming a detection period of 20 ns and the target is located at the position of 10 ns, the temporal distribution of reflected signal can be written as $S = h(t - t_0)$, while $h(t)$ is a Gaussian distribution with a $4\sigma = 1$ ns, σ is the standard deviation of the distribution and $t_0 = 10\,ns$. Moreover, $\mu_p = \int_0^T h(t - t_0)dt$ is the mean photon number of the reflected signal. Considering the existence of background noise, the total flux received by the LiDAR can be expressed as $F = h(t - t_0) + \mu_n$. In Fig. S21(a) and (b), we show the average photon number distribution of signal and noise, respectively. And μ_p is set as 1.5, μ_n is set as 0.0001. Based on the distribution, we can calculate the probability of detecting an event in each time bin, and the width of time bin is set as 1 ns, which is same with the manuscript. It Fig. S21(c), (d) and (e), we provide the detection probability $\sum_{k=N}^{+\infty} p(k)$, where N is 1, 2, 3, respectively. Here, $p(k) = \sum_{m=0}^{k} p_p(m|t)p_n(k - m)$ is the convolution of signal and noise as defined in the manuscript. p_{p} and p_{n} are the Poisson distribution of signal photons and noise photons. And we also display the max detection probability in each figure, shown as the number of the peak.

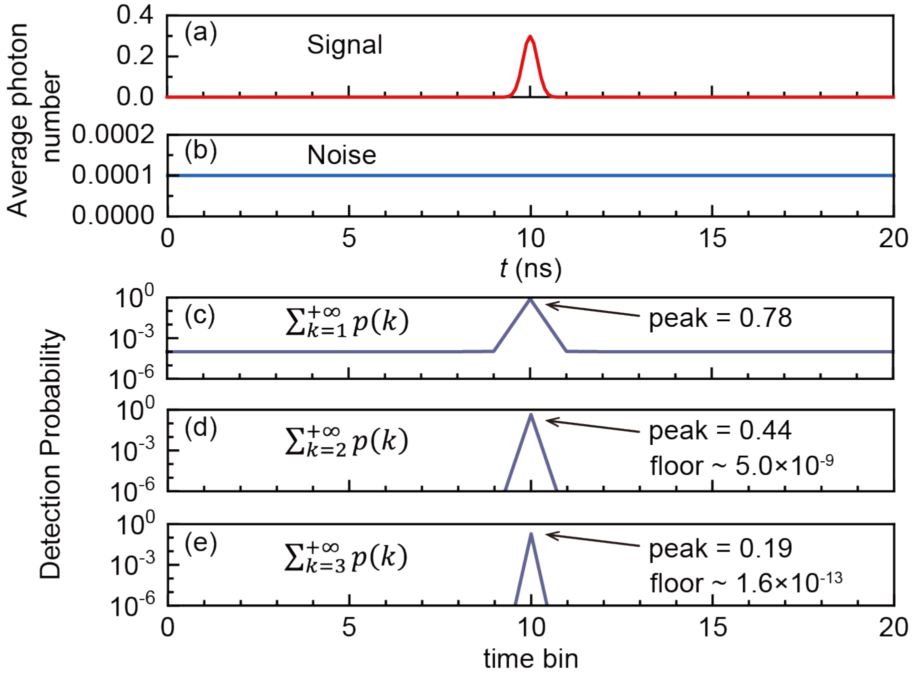

Fig. S21. The average photon distribution and detection probability. (a) The average photon number distribution of signal. (b) The average photon number distribution of noise. (c)-(e) The detection probability $\sum_{k=N}^{+\infty} p(k)$ in each time bin, where N is 1, 2, 3, respectively. The number of peaks gives the max probability under each condition.

Based on the detection probability, we can calculate the activation probability:

$$A = \frac{1 - (1 - \sum_{k=M}^{+\infty} p(k|t))^l}{1 - (1 - \sum_{k=N}^{+\infty} p(k|t))^l}. \quad (S10)$$

Here, we show the results when N is fixed to 1, M is 1, 2, 3 and l is 20 in Fig. S22(a). It can be seen that the activation probability is always 1 when M is equal to N. When M is increase to 2, the floor of the activation probability where no target locating is decreased to about 5×10^{-5}, and the peak remain 0.999. When M is 3, the peak is 0.986 and the floor is about 1.6×10^{-13}. Since the peak value is larger than 0.95, M=3 can satisfy the constraint of activation probability. 3 is also the max value of M which can satisfy the constraint. Therefore, N and M are set as 1 and 3 when μ_p is 1.5, respectively. In Fig. S22(b), we show the detection probability corrected by the activation probability. It can be seen that with the increase of M, the peak of signal where the target locating does not decrease a lot, but the noise floor is reduced. Thus, we can increase the SBR through APNF.

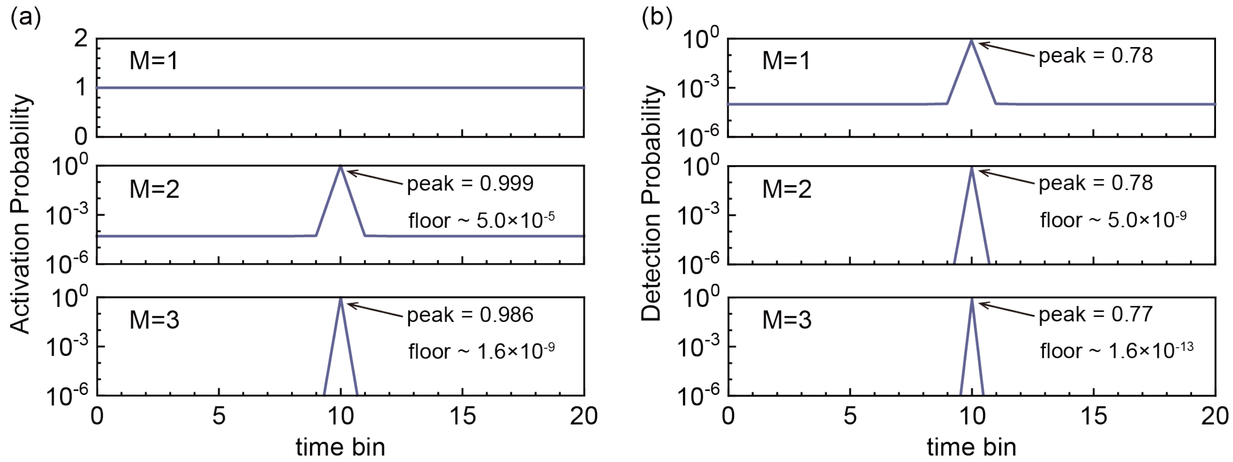

Fig. S22. The activation probability distribution and the corrected detection probability through APNF. (a) The activation probability distribution with different M. (b) The corrected detection probability distribution through APNF with different M.

In Fig. S23, we provide the original detected events with different photon numbers of one pixel in a 3D point cloud. The x-axis of the figure represents the flight time of detected photon events and the y-axis is the corresponding number of events. It can be seen that the results of one-photon contain a lot of chaotic events and it is difficult to accurately discriminate which are originated from the reflected signal. But there are no more chaotic events in the results of two-photon and three-photon. Since there is no four-photon event within this pixel, the M is determined to be 3. Then we use the flight time of three-photon events as the center time to construct time windows with duration time of 1 ns (the duration of the laser pulse) to filter the events of one-photon and two-photon. In Fig. S23(d), we show the results filtered by APNF. The events shown in Fig. S23(d) contain all one-photon, two-photon and three-photon events within the time windows. And most of noise events are filtered out after processed by APNF.

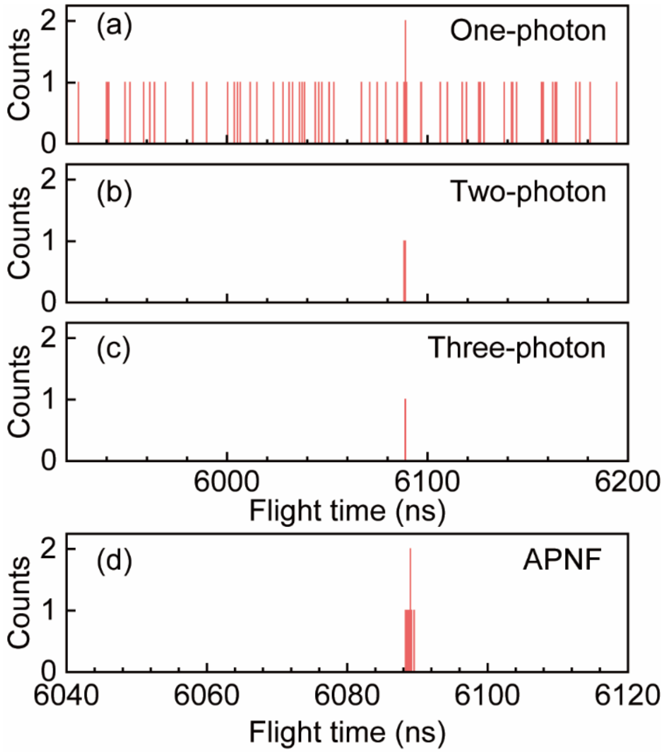

Fig. S23. The histograms of the flight time of recorded events in a pixel of a 3D point clouds. (a) The histogram of one-photon events. (b) The histogram of two-photon events. (c) The histogram of three-photon events. The x-axis of (a), (b) and (c) ranges from 5920 ns to 6200 ns. (d) The histogram of the filtered events through APNF when M=3. After processed by APNF, the remained events distribute between 6040 ns to 6120 ns.

Supplementary Note 13: Details of SNR

Figure. S24 gives the details of SNR_{APNF} and $SNR_{on/off}$ divided by SNR_Q. With the increase of μ_p, the quotient of $SNR_{on/off}$ divided by SNR_Q decreases rapidly. And the SNR_{APNF} / SNR_Q is almost always greater than -1 dB, which indicates the performance of proposed method to approach the quantum limited sensitivity.

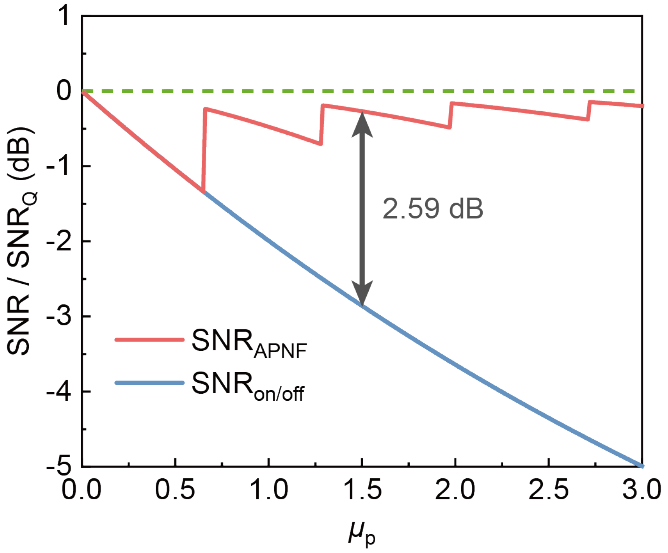

Fig. S24. The quotient of SNR_{APNF} and $SNR_{on/off}$ divided by SNR_Q. It can be seen that $SNR_{on/off}$ / SNR_Q decreases rapidly with the increase of μ_p, while SNR_{APNF} / SNR_Q is almost always larger than -1 dB. And the later one is 2.59 dB larger than the former when μ_p is 1.5.

Supplementary Note 14: Experimental Fisher information

Since the SNSPD we use in the experiment resolves the photon number through spatial multiplexing, it may fail to output the real photon number. Therefore, we calibrate the positive-operator-valued measure (POVM) elements of the detector at the first [4]. Since the detector is insensitive to the phase of the incident light, its POVMs only contains diagonal elements:

$$\pi_n = \sum_{i=0}^{\infty} \theta_i^{(n)} |i\rangle\langle i| \quad (S11)$$

where $\theta_i^{(n)}$ gives the probability of outcome *n* when i photons are absorbed by the detector. Consider an incident light with a distribution of *F*, the distribution *P* of the outcome of the detector can be written as:

$$P = F\Pi \quad (S12)$$

Here, Π is a $M \times N$ matrix, where a row of Π indicates distribution of *N* outcomes and a column of it represents the diagonal elements of π_n. The task of the calibration is to solve the matrix Π form known *P* and *F*. Figure. S25 show the experiment setup of the calibration. Here, a pulse laser with a repetition frequency of 500 kHz and a wavelength of 1550 nm is used as the source of coherent light. The emitted laser is transmitted by a single-mode fiber (SMF) to a calibrated optical attenuator. The attenuation of the attenuator can be adjusted by prepare 100 coherent states with different mean photon number per pulse. Based on the Poisson distribution, we can obtain a 100×10 matrix *F* which gives the statistic distribution of 0-9 photons for the 100 coherent states. The attenuated laser is then transmitted to the SNSPD through a multi-mode fiber (MMF). We here mainly focus on consider 0-click to 4-click since we detected up to 4-click in outdoor detection. The output of more than 4-click is regarded as a 4-click. The output of the detector is divided into four and then transmitted to a 4-channel threshold counter with thresholds of 1 to 4 to record the counts. The counts of 0-click to 4-click can be calculated from the counts of the 4 thresholds. Then, we can obtain a 100×5 matrix *P,* which gives the detected distribution of 0-click to 4-click. As we know *P* and *F*, the Π can be obtained by solving:

$$\min\{\|P - F\Pi\|_2 + g(\Pi)\} \quad (S13)$$

$$g(\Pi) = \epsilon \sum_{i,n} \left(\theta_i^{(n)} - \theta_{i+1}^{(n)}\right)^2 \quad (S14)$$

where $\epsilon = 0.1$ is the smoothing factor. This can be regarded as a convex optimization problem.

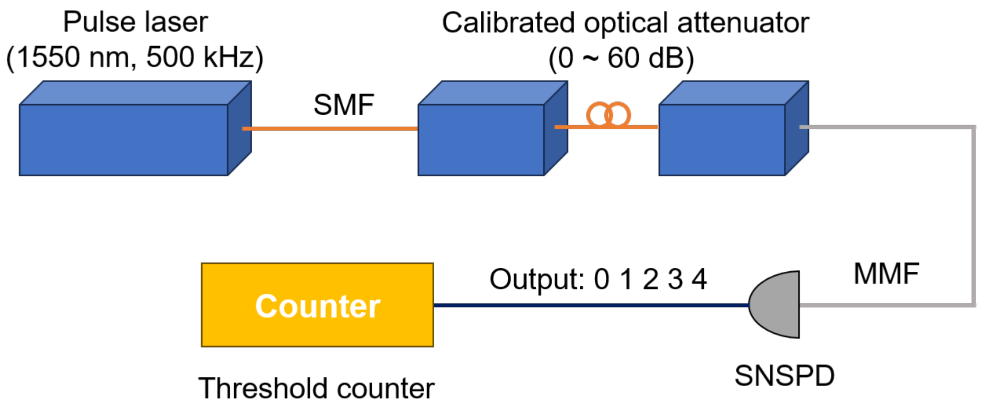

Fig. S25. The experimental setup of the detector calibration.

Figure. S26 shows the results of the calibration, which gives the $\theta_i^{(n)}$ versus i and n. Figure. S27 shows the original detected results of our detector in the experiment. The results deviate from the theoretical prediction as the photon number increases. Figure. S28 shows the results after correction by the POVM. Compared with the results shown in Fig. S27, it better meets the theoretical prediction. However, there are still some deviations when the mean photon number is large and the photon number is 4 since events with more than 4 photons start to be nonnegligible. In Fig. S29, we show the calculated Fisher information based on calibrated probabilities. To make it easy to read, we multiply the Fisher information by 1, 10, 100 and 1000. The results are consistent with the theoretical prediction.

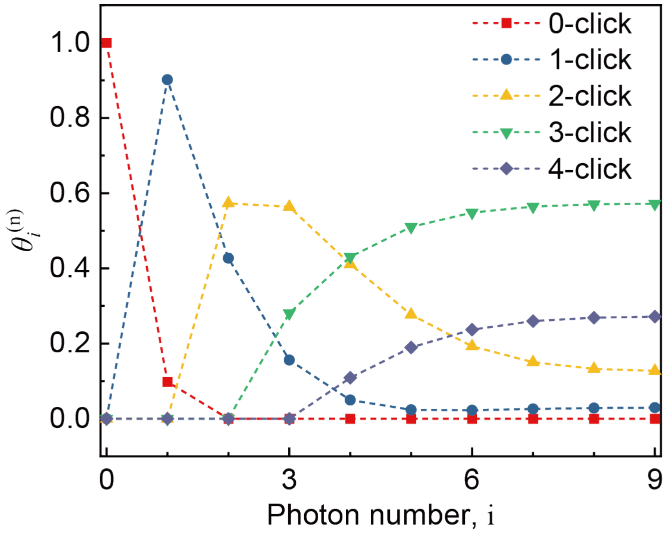

Fig. S26. Results of calibration of the detector.

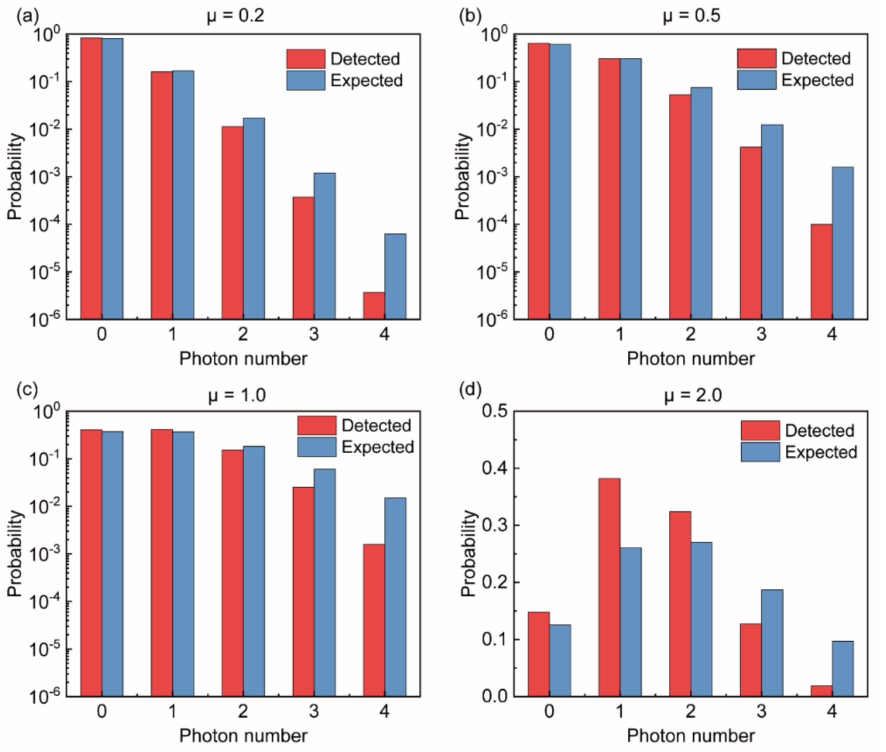

Fig. S27. Original detected photon-number distribution.

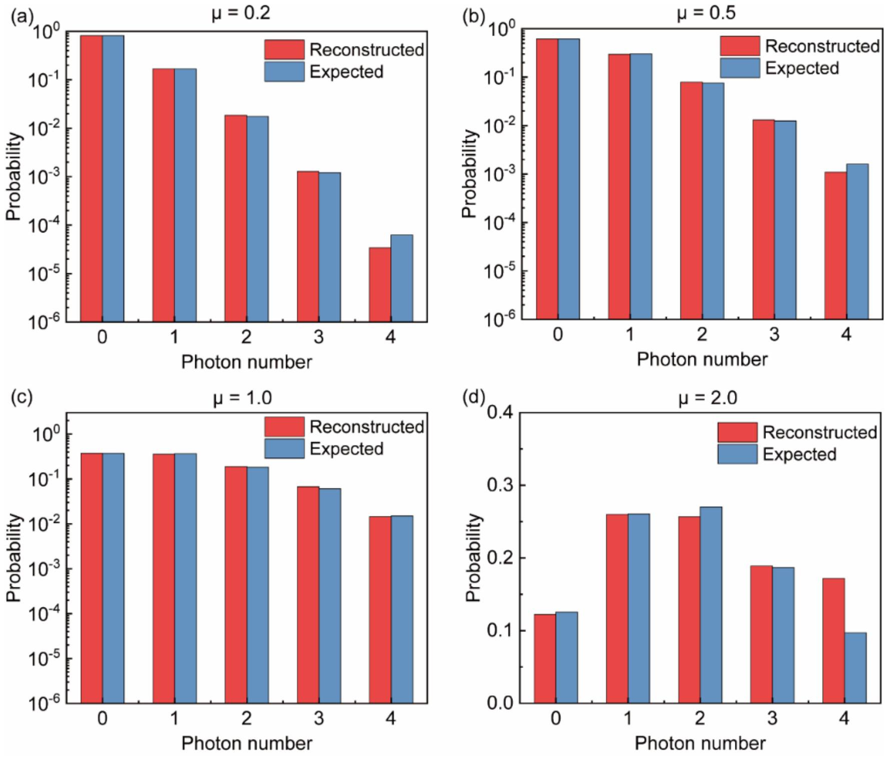

Fig. S28. Reconstructed photon-number distribution corrected by the POVM elements

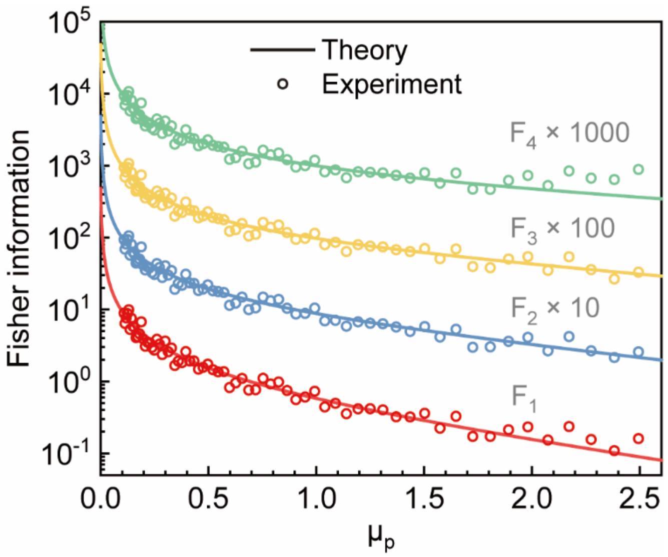

Fig. S29. Experimental Fisher information directly computed from the corrected probabilities.

Supplementary Note 15: Calibrating the statistical characteristics of background noise

To address the statistics of the noise light, we measure its second-order correlation, as shown in Fig. S30. The second-order correlation function of the background noise is approximately 1, which is not consistent with the bunching effect of single-mode thermal light. This indicates that the statistical distribution is more likely to be a Poisson distribution.

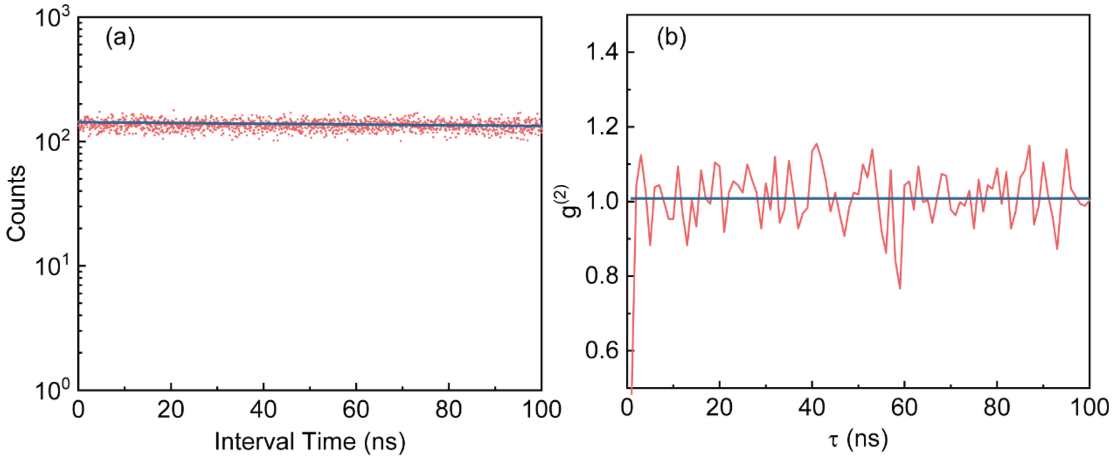

Fig. S30. The coincidence counts and 2nd-order correlation function $g^{(2)}$ of the background noise. (a) The coincidence counts of the background noise recorded by the Hydraharp 400. (b) The $g^{(2)}$ calculated from the coincidence counts.

Supplementary Note 16: Spatial distribution of *M*

In Fig. S31, we show the spatial distribution of *M* applied in APNF to process the data shown in Fig. 3 in the manuscript. In all areas shown in the graph, the value of *N* is 1. *M* with a value greater than 1 is mainly distributed on the main frame of the pylon, where the reflected signal is relatively strong. This indicates the capability of APNF to solve the target with reflectivity that is not uniform.

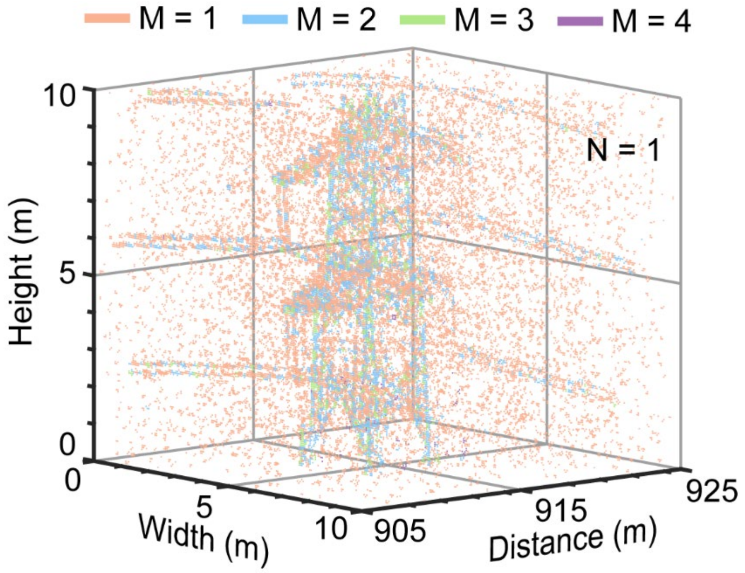

Fig. S31. The spatial distribution of *M*. *N* is always 1, and *M* greater than 1 is usually distributed on the main frame of the pylon.

Supplementary Note 17: Process data with different algorithms

One of the greatest advantages of APNF is that it can construct temporal gates with a duration time similar to the time jitter of the system, which depends on the pulse width of the laser and the time jitter of the detector, without prior knowledge and additional information. To further demonstrate the advantage of APNF, we enhance the noise in the dataset by generating random noise events according to the Poisson distribution through the Monte Carlo method. We further make comparisons between APNF and other denoising methods, as shown in Fig. S32. Here, we use a log-matched filter to process the on/off detection data, as shown in Fig. S32(a)-(c). When the noise intensity is original (10^5 counts per second), the structure of the pylon can be seen, but there are still many noise events. With the enhancement of the noise, the details of the target are gradually submerged by noise. In Fig. S32(d)-(f), we show the results processed by the Convex Optimal Deconvolution algorithm (Li) [1, 5], which uses a 3D spatiotemporal kernel to make a deconvolution. It can include the correlations between reflectivity and depth in the optimization. However, it fails to unmix the signal and noise when the noise is approximately 10^7 counts per second due to the failure in discriminating the reflectivity. Figure. S32(g)-(i) gives the results obtained by the photon-efficient algorithm (Shin) [6, 7], but it fails to display the details of the target and cannot work under heavy noise (10^7 counts per second). The "superpixel unmixing" algorithm (Rapp) [8] performs similarly to APNF under the original conditions, as shown in Fig. S32(j). This algorithm constructs superpixels according to the similarity of the reflectivity of adjacent pixels. However, it may build a false gate when the noise is large enough to submerge the differences between the background and the target, as shown in Fig. S32(k)&(l). The results of APNF display almost no noise even when the noise is enhanced by 20 dB, as shown in Fig. S32(m)-(o). This indicates the ability of APNF to extract signals under strong noise.

In Fig. S33, we show the corresponding depth images reconstructed from the 3D point clouds shown in Fig. S32.

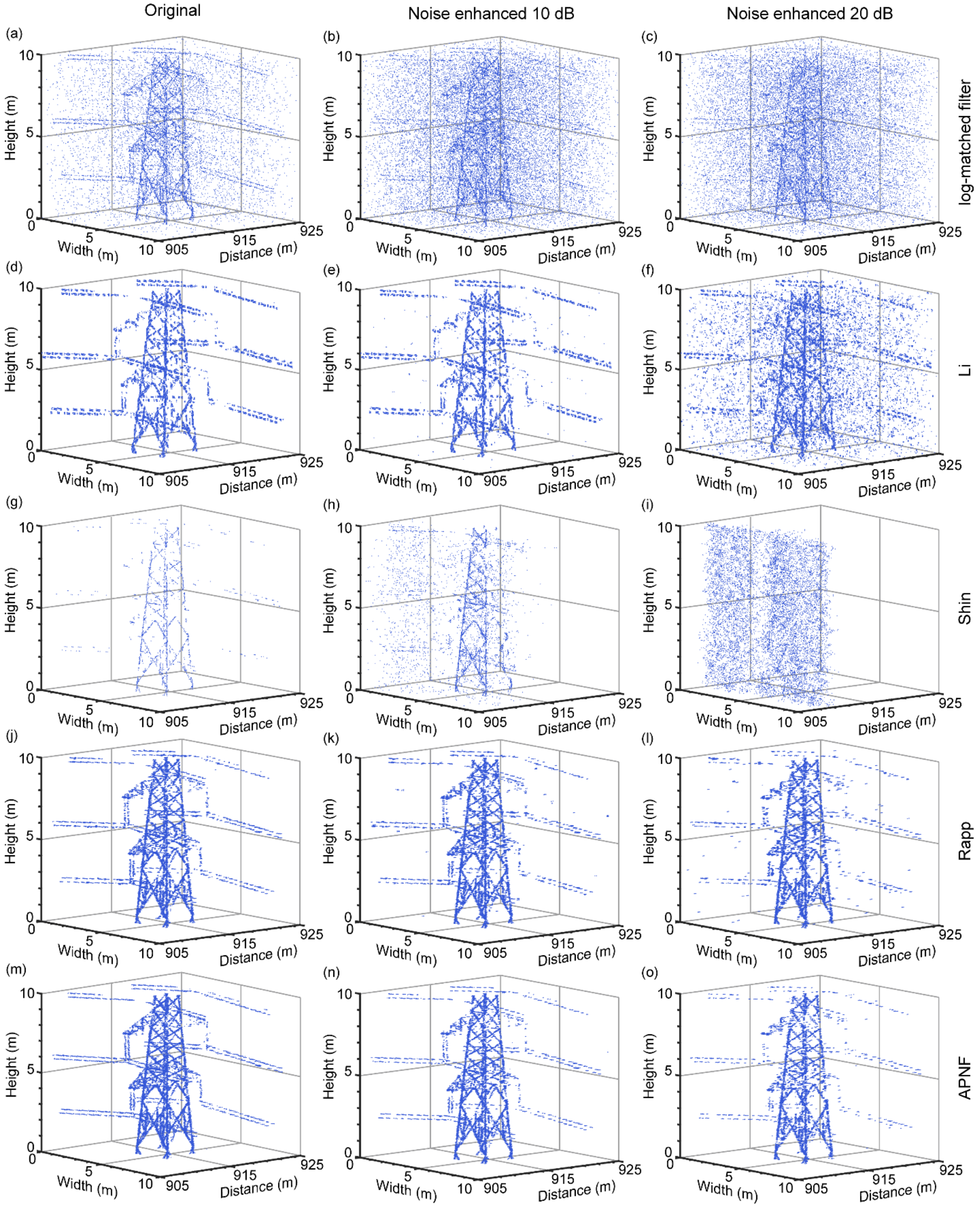

Fig. S32. The 3D point clouds processed by different denoising methods under 10^5, 10^6 and 10^7 noise counts per second. (a)-(c): The results obtained by the log-matched filter. (d)-(f): The point clouds processed by the Convex Optimal Deconvolution algorithm (Li). (g)-(i): The point clouds

processed by the photon efficient algorithm (Shin). (j)-(l): The point clouds processed by the "superpixel" algorithm (Rapp). (m)-(o): The point clouds processed by the proposed APNF.

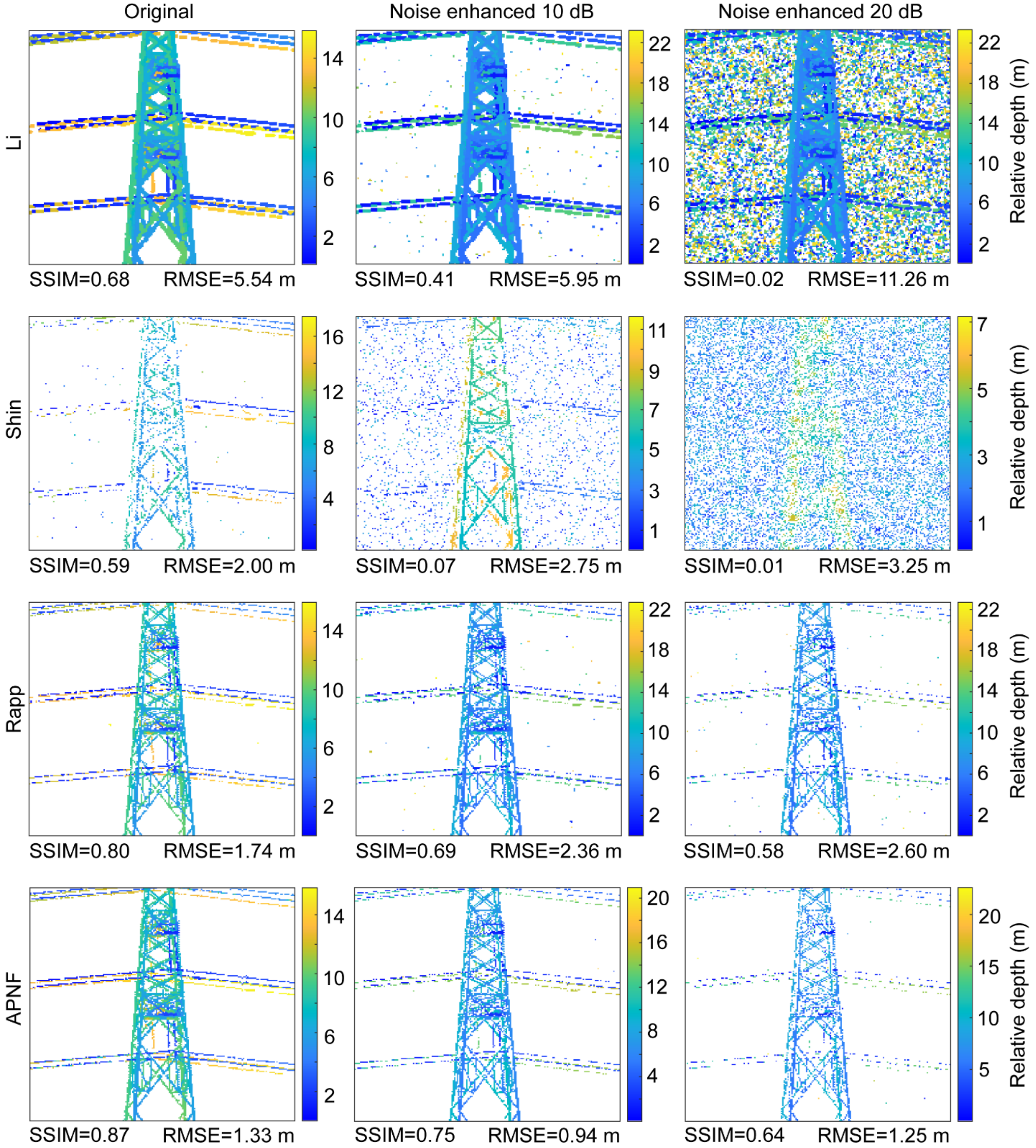

Fig. S33. The depth images reconstructed from the 3D point clouds are shown in Fig. S20.

Supplementary Note 18: Results of point cloud processing under free-running mode

Figure. S34 gives the results of point cloud processing under free-running mode. Without global gate, it can be seen that there are more noise and the target is submerged by the noise when using on/off detection. And the APNF bring an advantage of 42.35 dB on the SBR. The structure of the pylon can be seen after processed by APNF. Moreover, another target, a treetop, can also be extract with APNF. It indicates the ability of proposed method for multi-target detection, while applying global gate will loss the target.

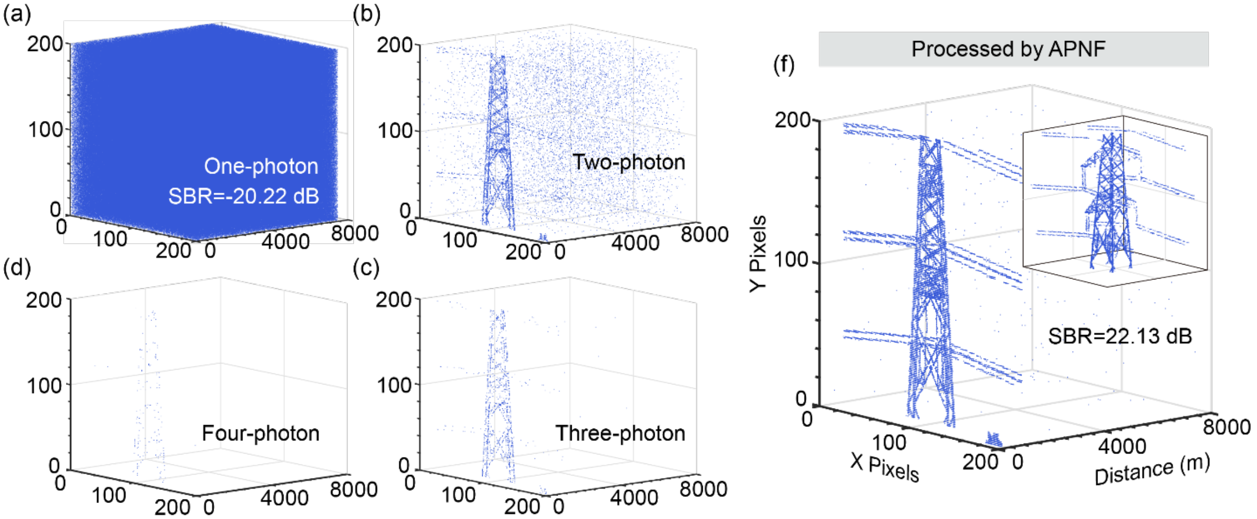

Fig. S34. The results of point cloud processing under free-running mode of LiDAR without global gating.

Supplementary Note 19: First-photon imaging using different photon number thresholds

Instead of on/off detection, we use photon number threshold detection [9] to achieve a larger SBR and then reconstruct reflectivity and depth images of the target based on first-photon imaging [10]. With the increase in the photon number threshold, the quality of reconstruction gradually improves, especially for reflectivity images. Due to the relatively small number of three-photon signals, the reconstruction of depth images has not achieved ideal results. However, it can still provide us with information about the target.

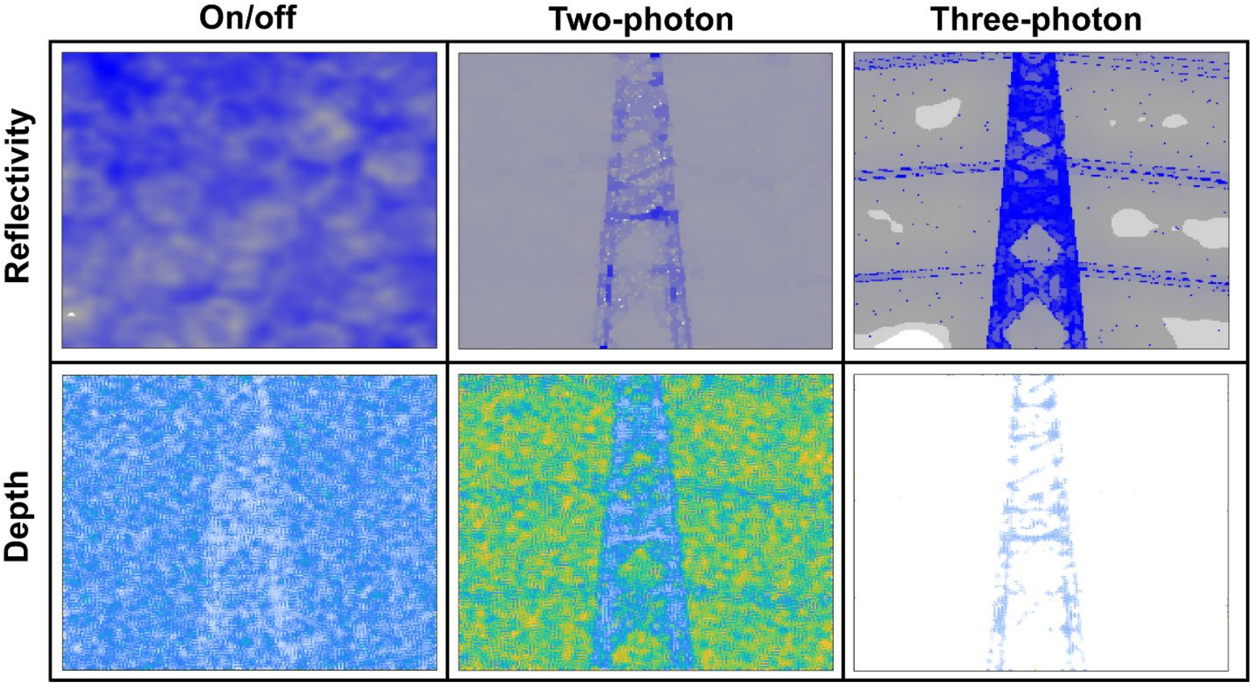

Fig. S35. Results of first-photon imaging using the results of different photon number threshold detections.

Supplementary Note 20: Parameters of the optical system

The system used in this paper is a common optical axis scanning system, and its specific parameters are as follows:

Table. S1. Main parameters of the optical system

<table>
<tr><td colspan="20">Laser</td></tr>
<tr><td colspan="5">Index</td><td colspan="5">Min</td><td colspan="5">Typical</td><td colspan="5">Max</td></tr>
<tr><td colspan="5">Wavelength (nm)</td><td colspan="5">1549</td><td colspan="5">1550</td><td colspan="5">1551</td></tr>
<tr><td colspan="5">Pulse Width (ns)</td><td colspan="5">1</td><td colspan="5">-</td><td colspan="5">7</td></tr>
<tr><td colspan="5">Repetition Frequency (kHz)</td><td colspan="5">1</td><td colspan="5">10</td><td colspan="5">100</td></tr>
<tr><td colspan="5">Pulse Energy (μJ)</td><td colspan="5">-</td><td colspan="5">14</td><td colspan="5">-</td></tr>
<tr><td colspan="5">Beam Quality (M^2)</td><td colspan="5">-</td><td colspan="5">-</td><td colspan="5">1.2</td></tr>
<tr><td colspan="20">Collimator</td></tr>
<tr><td colspan="10">Index</td><td colspan="10">Parameter</td></tr>
<tr><td colspan="10">Wavelength</td><td colspan="10">1550 nm</td></tr>
<tr><td colspan="10">Spot Diameter</td><td colspan="10">3.6 mm</td></tr>
<tr><td colspan="10">Divergence Angle</td><td colspan="10">0.032°</td></tr>
<tr><td colspan="10">NA</td><td colspan="10">0.15</td></tr>
<tr><td colspan="10">Focal Length</td><td colspan="10">18.75 mm</td></tr>
<tr><td colspan="20">Transmitter</td></tr>
<tr><td colspan="4">Magnification</td><td colspan="4">Input Aperture</td><td colspan="4">Output Aperture</td><td colspan="4">Input Angle</td><td colspan="4">Output Angle</td></tr>
<tr><td colspan="4">$10^{\times}$</td><td colspan="4">3.6 mm</td><td colspan="4">36 mm</td><td colspan="4">0.032°</td><td colspan="4">0.1 mrad</td></tr>
<tr><td colspan="20">Receiver</td></tr>
<tr><td colspan="10">Index</td><td colspan="10">Parameter</td></tr>
<tr><td colspan="10">Receiving Angle</td><td colspan="10">0.094 mrad</td></tr>
<tr><td colspan="10">Focal Length of Objective Lens</td><td colspan="10">320 mm</td></tr>
<tr><td colspan="10">Aperture</td><td colspan="10">60 mm</td></tr>
<tr><td colspan="10">Coupling efficiency to MMF</td><td colspan="10">>87%</td></tr>
<tr><td colspan="20">Fast Steering Mirror</td></tr>
<tr><td colspan="10">Index</td><td colspan="10">Parameter</td></tr>
<tr><td colspan="10">Reflectivity (@ 800 nm)</td><td colspan="10">98%</td></tr>
<tr><td colspan="10">Effective Aperture</td><td colspan="10">60 mm</td></tr>
<tr><td colspan="10">Deflection Angle</td><td colspan="10">±1.5°</td></tr>
<tr><td colspan="10">Angular Resolution</td><td colspan="10">2 μrad</td></tr>
</table>

Supplementary Note 21: Performances of the SNSPD

The SNSPD array used in this experiment has 16 pixels. Each pixel can approach a quantum efficiency of 100%, as shown in Fig. S36. At the working point we select, the quantum efficiency is ~90%. Considering the loss of photons during coupling, transmitting and absorption, the total system detection efficiency is ~45% at the selected working point, with total dark counting rates approximately 1600 cps (counts per second), as shown in Fig. S37(a). And the time jitter of the detector is 47.2 ps, as shown in Fig. S37(b).

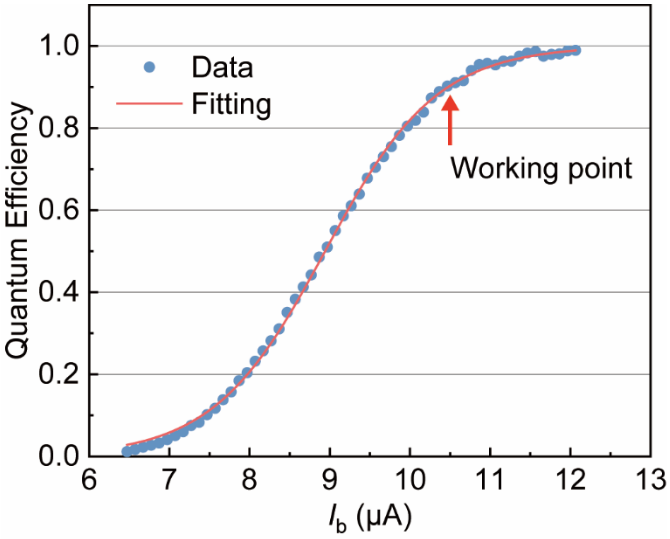

Fig. R36. The quantum efficiency of a pixel of the SNSPD. The working point is set to be 10.5 μA, where the quantum efficiency is ~90%.

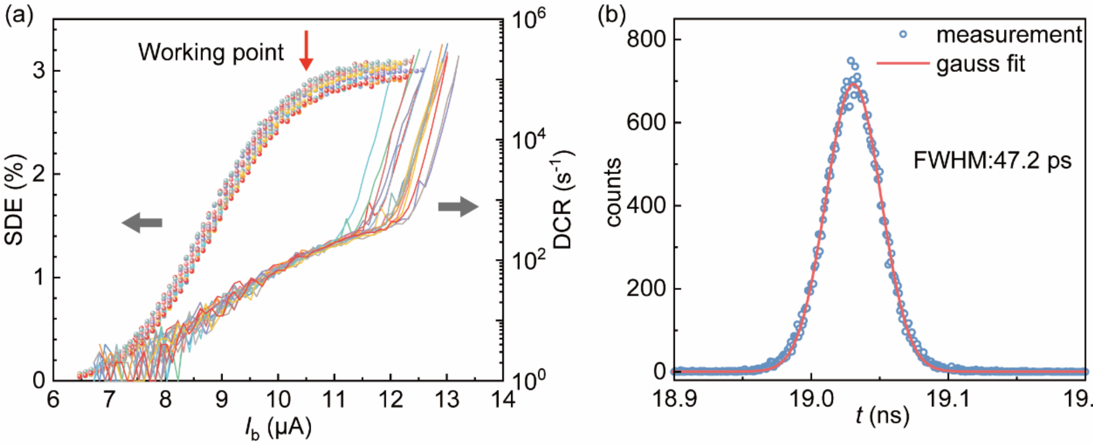

Fig. S37. The system detection efficiency (SDE), dark counting rate (DCR) and time jitter of the SNSPD. (a) The SDE and DCR of the SNSPD. At the working point, the total SDE is ~45% considering the loss of photons during coupling, transmitting and absorption, with total DCR approximately 1600 cps (counts per second). (b) The time jitter of the detector is 47.2 ps.

Supplementary Note 22: Theoretical performance of depth estimation

Different from reflectivity estimation, depth estimation is less determined by the quantum fluctuation of photons, but mainly determined by the shape of laser pulse and the properties of detector, such as timing jitter, dead time and response time.

In this work, the depth of target is estimated through maximum-likelihood estimation after the point cloud is processed by APNF. The estimation process can be expressed as:

$$\hat{\tau} = \arg\max_{\tau} L(\tau) = \arg\max_{\tau} \sum_{i=1}^{m} \log f(t_i - \tau) \qquad (S15)$$

where $L(\tau) = \log \prod_{i=1}^{m} f(t_i - \tau)$ is likelihood function of the probability density distribution (PDF) $f(t_i - \tau)$ of the detection time t_i, and m is the number of detected events. Since the illumination laser pulse is a Gaussian pulse, we can get $\hat{\tau} = \sum_{i=1}^{m} t_i / m$, and the estimated depth is $\hat{z} = c\hat{\tau}/2$. Then we calculate the Fisher information of the measurement:

$$\begin{aligned} F(\tau) &= \mathbb{E}\left[\left(\frac{\partial L(\tau)}{\partial \tau}\right)^2\right] \qquad (S16) \\ &= \mathbb{E}\left\{\left[\sum_{i=1}^{m} \frac{\partial}{\partial \tau} \log f(t_i - \tau_\Delta)\right]^2\right\} \\ &= \mathbb{E}\left\{\left[\sum_{i=1}^{m} \frac{\dot{f}(t_i - \tau_\Delta)}{f(t_i - \tau_\Delta)}\right]^2\right\} \\ &= \sum_{\Delta=1}^{T_r} \sum_{i=1}^{m} \left[\frac{\dot{f}(t_i - \tau_\Delta)}{f(t_i - \tau_\Delta)}\right]^2 f(t_i - \tau_\Delta) \end{aligned}$$

For each measurement, we assume that there is at most 1 event, *m*=1. Then the Fisher information of one detection is:

$$F(\tau) = \sum_{\Delta=1}^{T_r} \frac{\dot{f}^2(t_1 - \tau_\Delta)}{f(t_1 - \tau_\Delta)} = \int_0^{T_r} \frac{\dot{p}^2(t - \tau_0)}{p(t - \tau_0)} dt \qquad (S17)$$

where $p(t - \tau_0)$ is the PDF of detected photons determined by the shape of laser pulse, the mean signal photon number per pulse, the efficiency and dead time of the detector and the detection method we adopting. Thus, in order to acquire the Fisher information, we first need to establish the model $p(t - \tau_0)$. Since the depth estimation is performed after the APNF process, we only focus on the 6 ns surrounding the target and ignore the interference of noise, rather than the entire repetition period of the laser pulse. Same with the manuscript, the Gaussian laser pulse $s(t)$ used to illuminate the target has a width (FWHM) of 1 ns. In Fig. S38, we show the normalized shape of the laser pulse. To meet with the experiment set up, the bin width of a time bin is 4 ps. The mean signal photon number in the laser pulse is $\mu_{\mathrm{p}} = \int s(t) dt$.

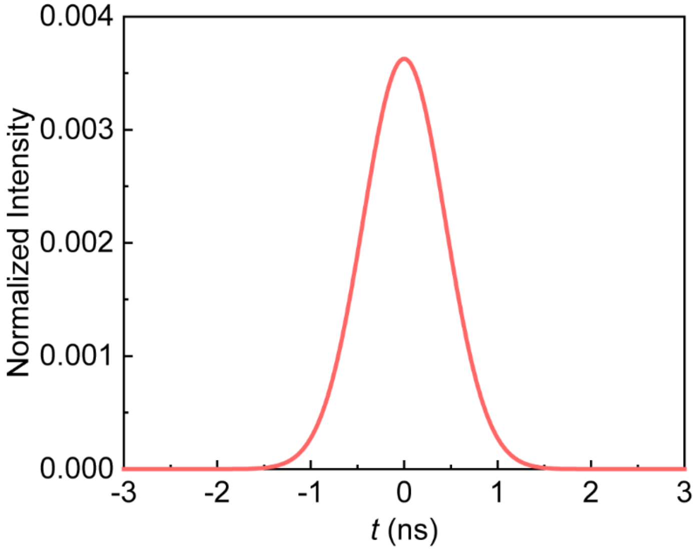

Fig. S38. The shape of the laser pulse used to illuminate the target.

Then we calculate the PDF of different detection method. For on/off detection, due to limitation of the dead time and the signal pulse duration time of the detector, it can only output one signal during the duration time of the laser pulse. Thus, we can't directly calculate the detection PDF just according to the intensity at a time bin when the condition $\mu_{\mathrm{p}} \ll 1$ is not meeting. Since the noise has been filtered out through APNF, we here assume that all output of the detector is caused by the signal laser pulse. The PDF of on/off detection can be calculated as:

$$p_{\mathrm{on/off}}(t) = \exp\left(-\int_{-T}^{t} s(\tau)\, d\tau\right) \times [1 - \exp(-s(t))] \qquad (S18)$$

That is the probability that the detector fails to be fired during [-*T*, t) and be fired at time *t*. Here *T* is set to be 3 ns. For PNR detection used in this work, it will be more complex to get the PDF, in which the detector can continuously respond to multiple photons. And *k* signals overlapping in the duration time of the rise edge will be regarded as a *k* photons event. The rise edge of the SNSPD is typically 1 ns. Thus, the PDF of PNR detection when *k* photons are detected can be expressed as:

$$p_k(t) = \sum_{0}^{k-1} \mathrm{poiss}\left(\int_{t-1ns}^{t} s(\tau)\, d\tau, m\right) \times \mathrm{poiss}[s(t), k-m] \qquad (S19)$$

where $\mathrm{poiss}(\mu, k) = \exp(-\mu) \times \mu^k / k!$ is the Poisson distribution.

For the maximum resolvable photon number N_0:

$$p_{N0}(t) = \sum_{m=0}^{N_0-1} \left\{ \mathrm{poiss}\left(\int_{t-1ns}^{t} s(\tau)\, d\tau, m\right) \times \left[1 - \sum_{n=0}^{N_0-m-1} \mathrm{poiss}(s(t), n)\right] \right\} \qquad (S20)$$

Here, we set N_0 to be 4. The model established here just aims to show the potential improvement of depth estimation brought by PNR, so it may not be comprehensive and perfect. A more comprehensive model about the detection process can be referred to the reference [11]. In Fig. S39,

we provide the PDF of on/off detection and different photon number detection with different μ_{p} in the laser pulse. From Fig. S39(a), we can see that the peak of on/off detection gradually shifts forward with the increase of μ_{p}, which is called the saturated stacking effect in single photon detection cause by the dead time of the detector. And its distribution also become narrow with the increase of μ_{p} due to the saturated stacking effect. It cannot be avoided in remote LiDAR especially those working outdoor, in which the intensity of echo signal varies in a large range. In Fig. S39(b), we show the PDF of PNR detection with different detected photons. With the increase of detected photon number, the PDF shifts afterward.

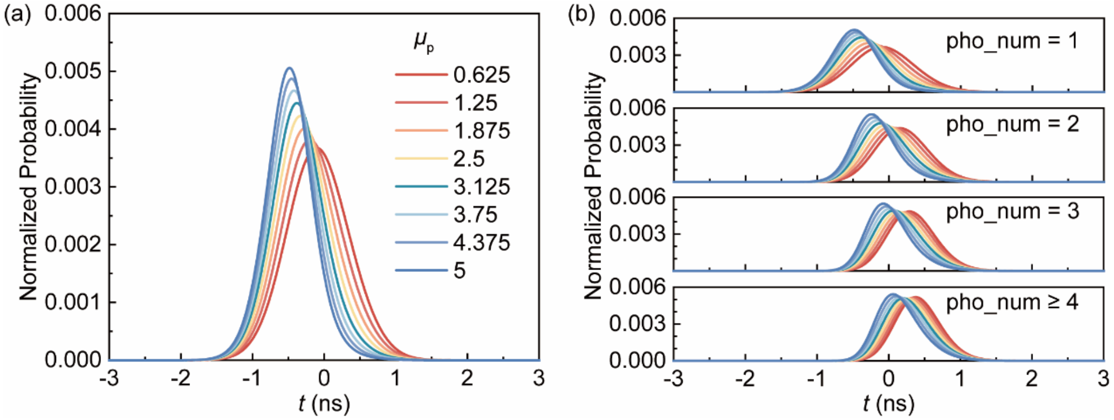

Fig. S39. The probability density function (PDF) of detected photons with different detection method with different mean signal photon number. (a) The PDF of on/off detection. (b) The PDF of different photon number.

The PDF of PNR detection is defined as the sum of the number weighted PDF of different detected photon numbers:

$$p_{\mathrm{PNR}}(t) = \sum_{k=0}^{N0} k p_k(t) \qquad (S21)$$

Here, the $p_k(t)$ is the poiss(μ_{p}, k) weighted result of normalized equation (S19) and (S20). In Fig. S40, we show the PDF of detected photons with PNR detection which can resolve up to 4 photons (PNR=4). It can be seen that the PDF first shifts afterward then forward with the increase of μ_{p}. This interference of saturated stacking effect still exists, but has been alleviated compared to Fig. S39(a).

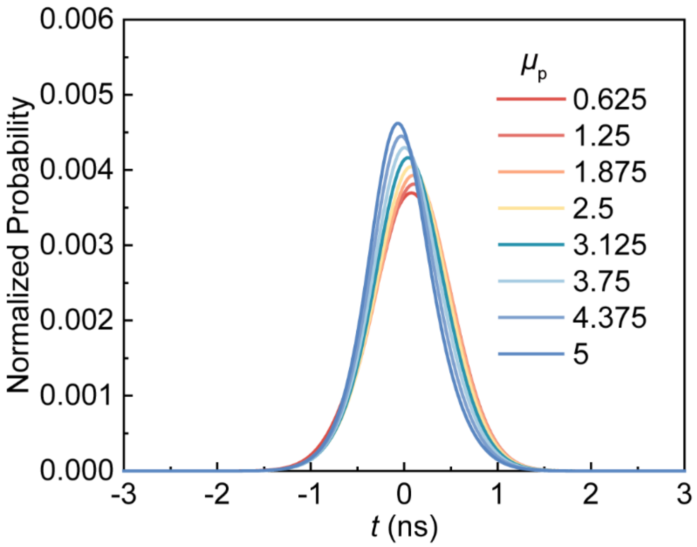

Fig. S40. The PDF of detected photons with PNR detection which can resolve up to 4 photons with different mean signal photon number.

Obtained the $p(t-\tau_0)$, we can then calculate the Fisher information $F(\tau)$. And the Cramer-Rao bound CRB $= 1/NF(\tau)$, where N is the total number of detected photons. For on/off detection, the mean detected photon number per emitted laser pulse is expected to be $1-\exp\,(\mu_\mathrm{p})$. Then N for on/off detection is $N = l \times (1-\exp\,(-\mu_\mathrm{p}))$, where l is the number of emitted laser pulses. And N for PNR detection is:

$$N = l * \left[\sum_{k=1}^{N_0-1} k \times \mathrm{poiss}(\mu_\mathrm{p}, k) + N_0\left(1 - \sum_{k=0}^{N_0-1} \mathrm{poiss}(\mu_\mathrm{p}, k)\right)\right] \qquad (S22)$$

In Fig. S41(a), we provide the expected CRB of depth estimation when $l = 1$ with on/off detection and PNR=4 detection. It can be seen that both the estimation variances of on/off detection and PNR=4 detection decrease with the increase of μ_p. For on/off detection, it is cause by the reduced distribution width of PDF and the improved probability of detecting an event. For PNR detection, though the distribution width of PDF does not decrease a lot with the increase μ_p, but it can detect more photon in a detection. Thus, the variance of PNR detection is lower than that of on/off detection. We also provide the mean square error (MSE) of depth estimation, as shown in Fig. S41(b). The MSE can be calculated as MSE $= \Delta^2 + d^2$, where d is the deviation of the mean value of the detection PDF to the true value. The MSE of on/off detection first decrease and then increase with the increase of μ_p. The decrease is caused by the decrease of the variance of estimation. And the increase is caused the deviation of the PDF due to the saturated stacking effect. Since the PNR detection is able to alleviate the interference of the saturated stacking effect as shown in Fig. S40, the MSE of it does not increase obviously than the variance.

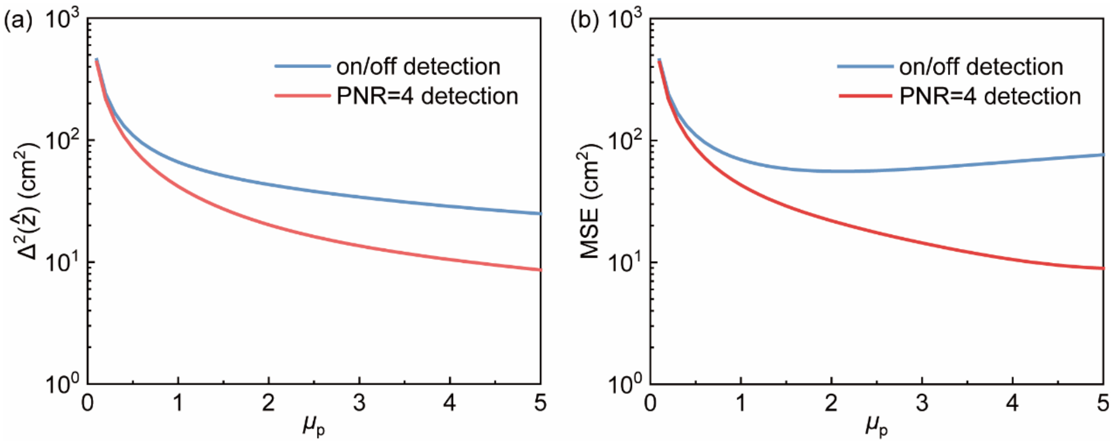

Fig. S41. The theoretical performance of depth estimation of on/off detection and PNR=4 detection with a single laser pulse emitted. (a) The CRB variance of depth estimation versus μ_{p}. (b) The MSE of depth image reconstruction versus μ_{p}.

Then, we investigate the performance of depth estimation with different detection method through Monte Carlo simulation experiment. First, a target with different reflected signal intensities is used to produce the detection event. As shown in Fig. S42, the mean photon number μ_{p} of reflected signal is set to be various from 0.6 to 4.8 with a step of 0.6. Moreover, the depth of the target is also various at different areas. In Fig. S43(a), we show the ground truth of the depth image and the depth is various from 16 cm to 32 cm with a step of 2 cm.

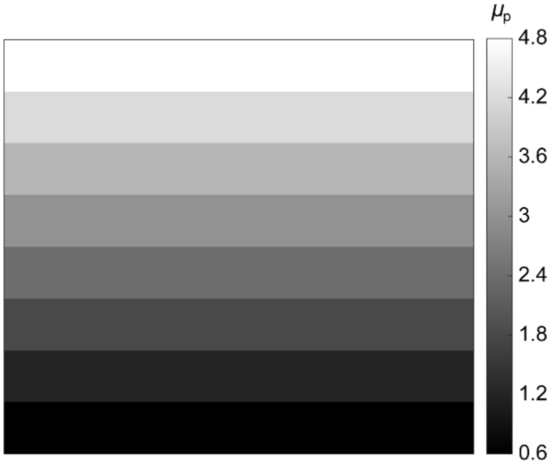

Fig. S42. The target has the different reflected mean photon number μ_{p} at different area. And μ_{p} is various from 0.6 to 4.8 with a step of 0.6.

Then, we use Monte Carlo method to generate the detection events with on/off detection and PNR=4 detection, respectively. And 20 laser pulse per pixel is assumed to be emitted, corresponding 20 measurements per pixel (mpp). Here, the pixel-wise maximum likelihood estimation is taken to reconstruct the depth images. In Fig. S43(c), it can be seen that the result of PNR detection is in consistent with the ground truth. However, the result of on/off detection

deviates from the ground truth when the reflected μ_p is large, as shown in Fig. S43(b). The same column of the three images is extract to display the details, as shown in Fig. S44. It can be seen that the results of PNR detection match the ground truth better than those of on/off detection. And the results of PNR detection also have a less fluctuation.

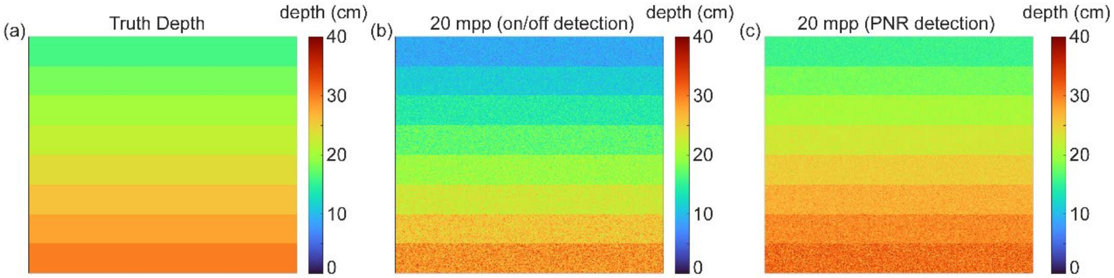

Fig. S43. The depth image. (a) The true depth image. (b) The estimated depth image with on/off detection. And 20 measurements per pixel (mpp) is taken here. (c) The estimated depth image with PNR=4 detection.

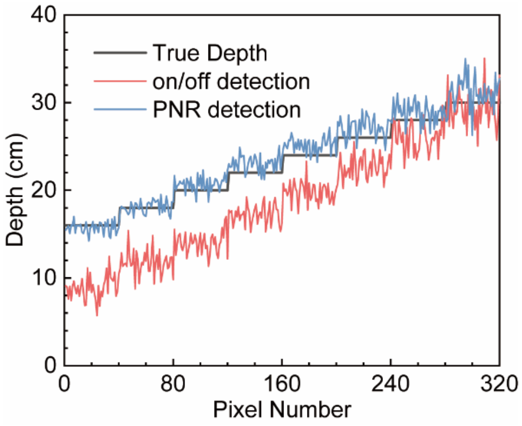

Fig. S44. The depth value of a same column of the three depth images shown in Fig. S43. It can be seen that the results of PNR detection match the ground truth better than those of on/off detection. And the results of PNR detection also have a less fluctuation.

We also calculate the simulated variances and MSE of the estimated images and compare them with theoretical value, as shown in Fig. S45. It can be seen that the simulated results are in consistent with the theoretical value. These results demonstrate the advantages of PNR detection for depth estimation in outdoor remote LiDAR detection. The improvement is brought by the alleviation of saturated stacking effect and the capacity of PNR detection to detect more photons in a measurement.

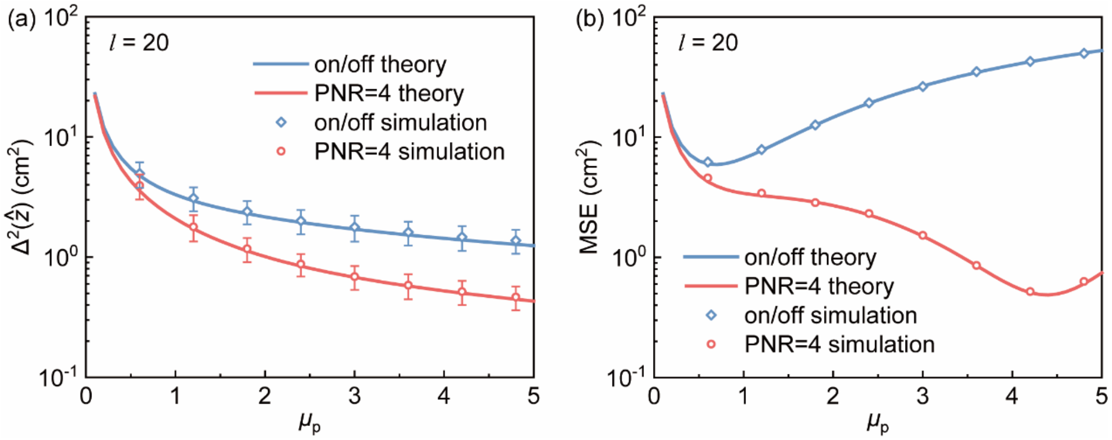

Fig. S45. The calculated variances and MSE of the estimated depth images. The solid lines represent the theoretical value, and the dots are calculated from the depth images shown Fig. S43(b) and (c), respectively.

References

1. Li Z-P, Ye J-T, Huang X, Jiang P-Y, Cao Y, Hong Y*, et al.* Single-photon imaging over 200 km. *Optica* 2021, **8**(3)**:** 344-349.

2. Bao F, Wang X, Sureshbabu SH, Sreekumar G, Yang L, Aggarwal V*, et al.* Heat-assisted detection and ranging. *Nature* 2023, **619**(7971)**:** 743-748.

3. DesAutels GL. A modern review of the johnson image resolution criterion. *Optik* 2022, **249:** 168246.

4. Schapeler T, Philipp Höpker J, Bartley TJ. Quantum detector tomography of a 2×2 multi-pixel array of superconducting nanowire single photon detectors. *Optics Express* 2020, **28**(22)**:** 33035-33043.

5. Li Z-P, Huang X, Cao Y, Wang B, Li Y-H, Jin W*, et al.* Single-photon computational 3D imaging at 45 km. *Photonics Research* 2020, **8**(9)**:** 1532-1540.

6. Shin D, Xu F, Venkatraman D, Lussana R, Villa F, Zappa F*, et al.* Photon-efficient imaging with a single-photon camera. *Nature Communications* 2016, **7**(1)**:** 12046.

7. Shin D, Kirmani A, Goyal VK, Shapiro JH. Photon-Efficient Computational 3-D and Reflectivity Imaging With Single-Photon Detectors. *IEEE Transactions on Computational Imaging* 2015, **1**(2)**:** 112-125.

8. Rapp J, Goyal VK. A Few Photons Among Many: Unmixing Signal and Noise for Photon-Efficient Active Imaging. *IEEE Transactions on Computational Imaging* 2017, **3**(3)**:** 445-459.

9. Cohen L, Matekole ES, Sher Y, Istrati D, Eisenberg HS, Dowling JP. Thresholded Quantum LIDAR: Exploiting Photon-Number-Resolving Detection. *Physical Review Letters* 2019, **123**(20)**:** 203601.

10. Kirmani A, Venkatraman D, Shin D, Colaco A, Wong FNC, Shapiro JH*, et al.* First-Photon Imaging. *Science* 2014, **343**(6166)**:** 58-61.

11. Rapp J, Ma Y, Dawson RMA, Goyal VK. High-flux single-photon lidar. *Optica* 2021, **8**(1)**:** 30-39.
